# Identifying systematic heterogeneity patterns in genetic association meta-analysis studies

**DOI:** 10.1371/journal.pgen.1006755

**Published:** 2017-05-01

**Authors:** Lerato E. Magosi, Anuj Goel, Jemma C. Hopewell, Martin Farrall, on behalf of the CARDIoGRAMplusC4D Consortium

**Affiliations:** 1 Wellcome Trust Centre for Human Genetics, University of Oxford, Oxford, United Kingdom; 2 Division of Cardiovascular Medicine, Radcliffe Department of Medicine, University of Oxford, Oxford, United Kingdom; 3 Nuffield Department of Population Health, University of Oxford, Oxford, United Kingdom; Emory University, UNITED STATES

## Abstract

Progress in mapping loci associated with common complex diseases or quantitative inherited traits has been expedited by large-scale meta-analyses combining information across multiple studies, assembled through collaborative networks of researchers. Participating studies will usually have been independently designed and implemented in unique settings that are potential sources of phenotype, ancestry or other variability that could introduce between-study heterogeneity into a meta-analysis. Heterogeneity tests based on individual genetic variants (e.g. *Q*, *I*^2^) are not suited to identifying locus-specific from more systematic multi-locus or genome-wide patterns of heterogeneity. We have developed and evaluated an aggregate heterogeneity *M* statistic that combines between-study heterogeneity information across multiple genetic variants, to reveal systematic patterns of heterogeneity that elude conventional single variant analysis. Application to a GWAS meta-analysis of coronary disease with 48 contributing studies uncovered substantial systematic between-study heterogeneity, which could be partly explained by age-of-disease onset, family-history of disease and ancestry. Future meta-analyses of diseases and traits with multiple known genetic associations can use this approach to identify outlier studies and thereby optimize power to detect novel genetic associations.

## Author summary

Meta-analysis of genome-wide association studies (GWAS) is a valuable tool for the discovery of genes that protect or predispose individuals to common complex diseases. It can though be hampered by excessive heterogeneity among its participating studies. To date, the impact of heterogeneity is assessed locally on an individual SNP basis using Q, I^2^ and τ^2^ statistics. Here, we present a new heterogeneity statistic, *M* that assesses genomic (multi-SNP) patterns of heterogeneity in GWAS meta-analysis with enhanced power compared to conventional methods. When applied to a recent GWAS meta-analysis of coronary artery disease, the new statistic revealed substantial patterns of systematic heterogeneity, much of which was attributed to differences in ancestry, age-of—disease onset and family history of disease. The new method can dissect genomic heterogeneity patterns to flag underperforming studies that could comprise the power of the meta-analysis as well as identify influential studies with advantageous design features to inform future meta-analyses of multifactorial disease.

## Introduction

The common disease—common variant (CD-CV) hypothesis has been confirmed by the discovery of thousands of robustly associated loci for a wide variety of complex diseases and quantitative inherited traits [1]. The genetic effects conferred by common susceptibility loci tend to be small (per-allele disease odds ratios < 1.2 or trait variance < 0.2%) [2] with the consequence that they are frequently only reliably detected in association studies based on upwards of tens of thousands of individuals. Such large sample sizes require considerable resources to complete the necessary participant recruitment, phenotyping and genotyping, resources that are unlikely to be available to individual research groups.

In response, collaborative networks of researchers have formed consortia in order to assemble large collections of genome-wide association data [3]. Participating studies that were independently commissioned are likely to include specific and varied design features, for instance the precise specification of the phenotype or ascertainment criterion, environmental risk factor profiles or genetic ancestry. These sources of variation could influence the meta-analysis and introduce genetic heterogeneity of effect sizes between participating studies, which would reduce power to detect an overall genetic association. Heterogeneity analysis is currently performed on a variant-by-variant basis, which is potentially sensitive to locus-specific effects, for example specific gene-environment interactions that affect a minority of contributing studies. Furthermore, as the true effect sizes of genetic associations tend to be small with relatively large variances at the individual study level, single variants contain modest information on systematic between-study heterogeneity. Together, these features might mask outlier studies in a meta-analysis that show systematic patterns of heterogeneity due to design features that affect the majority of the associated variants. For example, many diseases have a variety of clinical presentations that could affect the case-mix under alternative recruitment frameworks. In multi-ethnic stroke meta-analysis, the distribution of ischaemic and haemorrhagic cases might differ among populations [4]. Furthermore, sub-phenotypes of disease might have larger or smaller genetic components. For example, although the majority of coronary artery disease (CAD) associated loci showed similar effect sizes in analyses based on the subset of cases with myocardial infarction alone versus a broader CAD phenotype (coronary stenosis >50%, acute coronary syndrome and chronic stable angina), discrepant effect sizes were evident at the *HDAC9* and *ABO* loci [3]. Moreover, sampling patients with younger or older age-of-onset of disease or with or without a family-history of disease could affect genetic risk profiles according to the multifactorial liability threshold model [5].

We have therefore developed an analytic approach to search for systematic between-study heterogeneity patterns in genetic association meta-analysis projects. Our approach builds upon the established random-effects meta-analysis method [6], to combine information from multiple genetic variants into an integrated heterogeneity statistic. We first assess the analytic power of the new method to compare its performance with a conventional method to detect heterogeneity and then confirm the size and further explore the power of the new method in computer simulation exercises. Finally, we apply the method to a recent GWAS meta-analysis of CAD [3].

## Results

### Size and power of the aggregate heterogeneity statistics

To empirically assess the theoretical distributions of *M*, *SPRE* statistics for 2, 10, 25 or 50 variants were randomly sampled from a Φ(0, 1) distribution in 10,000 replicates to approximate the null hypothesis of no systematic heterogeneity. The empirical and theoretical distributions of *M* match very closely irrespective of the number of variants (S1 Fig and S1 Table).

**Fig 1:**
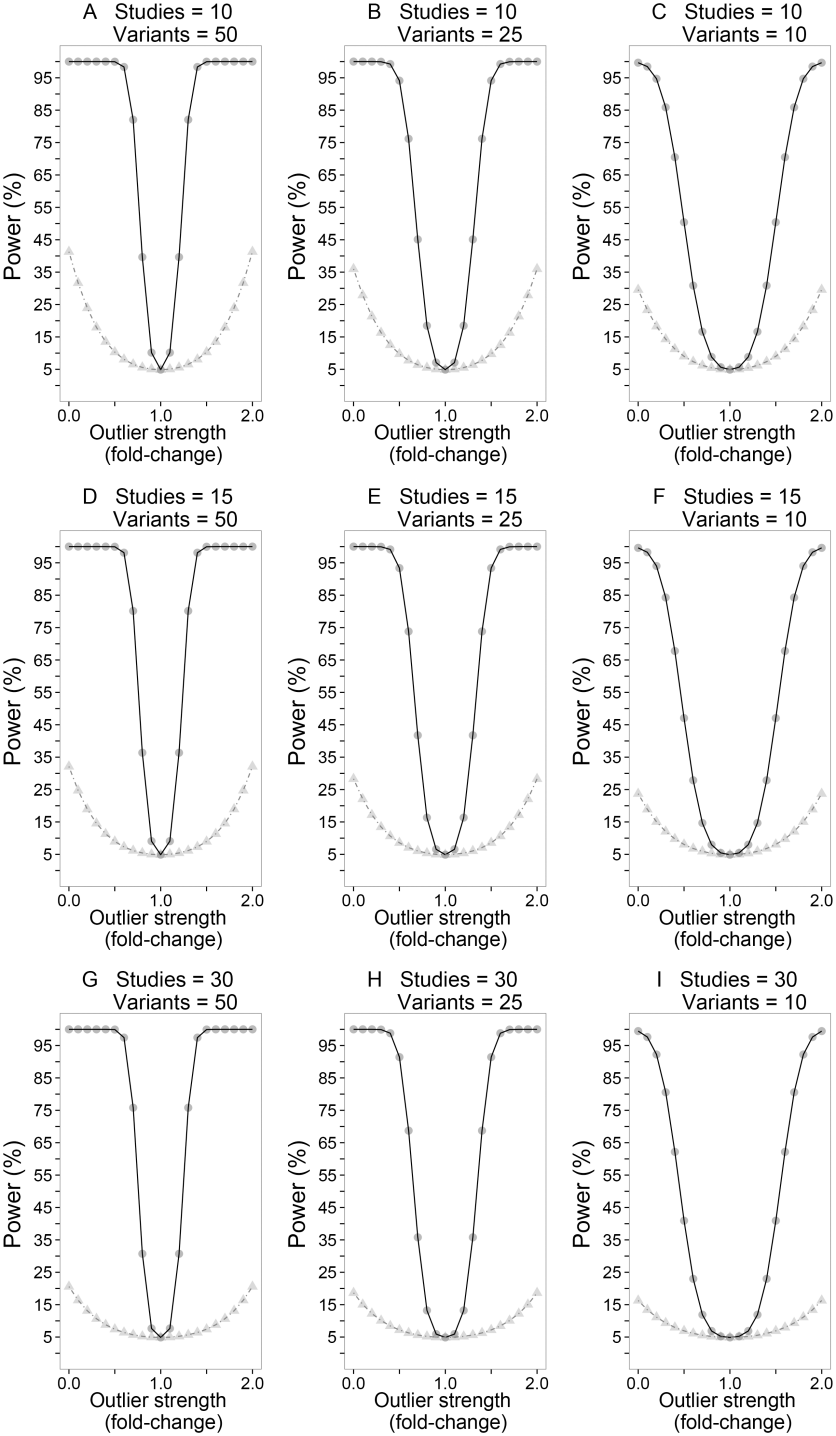
A comparative power analysis of *M* and Cochran’s Q to detect systematic heterogeneity. The nine panels show (from left to right) simulations for 10, 15 and 30 studies, examined at 50, 25 and l0 variants; Data points for the *M* statistic are represented by filled circles whilst those for Cochran’s Q are denoted by filled triangles. Each data point represents a meta-analysis scenario where effect sizes for the non-outlier studies were held constant (log_e_(odds ratio) = 0.182 i.e. odds ratio = 1.2) to model homogeneous effects. The effect sizes of variants in the outlier study were the product of the non-outlier effect size (i.e. log_e_(odds ratio) = 0.182) and a parameter (fold-change) to model a continuous series of systematic heterogeneity patterns. All studies were equally weighted (standard error of log_e_(odds ratio) = 0.1).

The analytic power of *M* to detect heterogeneity was compared with Cochran’s *Q* statistic [7, 8], a method that is routinely used to detect heterogeneity in meta-analyses and also underpins the *I*^*2*^ inconsistency index [9]. Multiple testing of *V* variants (for *Q*) and *S* studies (for *M*) was allowed for by applying Bonferroni’s adjustment to ensure that the family-wise error rates (FWER) for each method were equally controlled. Fig 1 shows the comparative power for 10, 25 and 50 variants in 10, 15 and 30 studies; the effect sizes for the *S*-1 “non-outlier” studies were held constant (log_e_(odds ratio) = 0.182 i.e. odds ratio = 1.2) to model homogeneous effects. The effect sizes for the variants in the outlier study were the product of the “non-outlier” effect size (i.e. log_e_(odds ratio) = 0.182) and a parameter (fold-change) to model a continuous series of systematic heterogeneity patterns. All studies were equally weighted (standard error of log_e_(odds ratio) = 0.1). It is clear that under all scenarios examined (Fig 1), that *M* had greater power than *Q* to detect systematic heterogeneity patterns. The power of *M* to detect systematic heterogeneity increased as the fold-change parameter differed from 1 as well as with larger numbers of variants but was slightly attenuated as the number of studies (and multiple testing burden) increased.

We then used Monte-Carlo computer simulations to empirically assess the type 1 and 2 error rates in a more complex series of “real world” meta-analysis scenarios for differing numbers of variants and studies. Variants were modelled to confer disease risks of varying magnitudes (S2 Table); the effect size distribution across the variants was inspired by an overview of GWAS findings [10], which documented the well-established pattern of a progressively larger number of variants with smaller effect sizes. Random variation in effect sizes for the variants in different studies was included by sampling the effect sizes (i.e. *β* coefficients scaled as log_e_(odds ratio)) from a Φ(*β*, *σ* = 0.10) distribution; this induces a background random heterogeneity pattern that affects all studies upon which we attempt to detect an overlying systematic heterogeneity pattern that only affects a single outlier study. Studies were assigned equal weights in the meta-analysis by fixing the standard errors of the simulated effect sizes based on the median value of standard errors for variants in a recent GWAS meta-analysis [3] (i.e. *SE*(*β*) = 0.08). Thus each variant was modelled with a background heterogeneity inconsistency index [11] *I*^2^ = 100 × 0.10^2^ / (0.10^2^ + 0.08^2^) = 60.5%. Table 1 presents empirical type 1 error rates derived from 1,000 replicates to compare with Bonferroni corrected asymptotic p-values < 0.05. The type 1 errors for the *M* statistics were mostly conservatively controlled in these simulation scenarios.

**Table 1:** Empirical type- 1 error rates and power to detect an outlier study for *M* at threshold α = 0.05.

# Studies	# Variants	Type- 1 error	Power
30	50	0.050	98.9
	25	0.054	98.1
	10	0.056	39.9
15	50	0.033	99.2
	25	0.034	97.6
	10	0.039	40.3
10	50	0.024	98.7
	25	0.033	96.7
	10	0.025	35.7

GWAS meta-analysis simulation experiments each based on 1000 replicates. Studies were equally weighted (i.e. *SE*(*β* coefficients) = 0.08). Variant effect sizes for studies in the type-1 error analysis were sourced from an L-shaped distribution (S2 Table). In the power analysis, variant effect sizes for studies showing typical effects were sourced from S2 Table whilst effect sizes for variants in the outlier study were calculated as a multiple of the typical effect size. For example, effect sizes for variants in an outlier study 2-fold-stronger than studies showing typical effects would be computed as (2 x ({0.04, 0.12, 0.2, 0.28, 0.4}, σ = 0.10).

**Fig 2:**
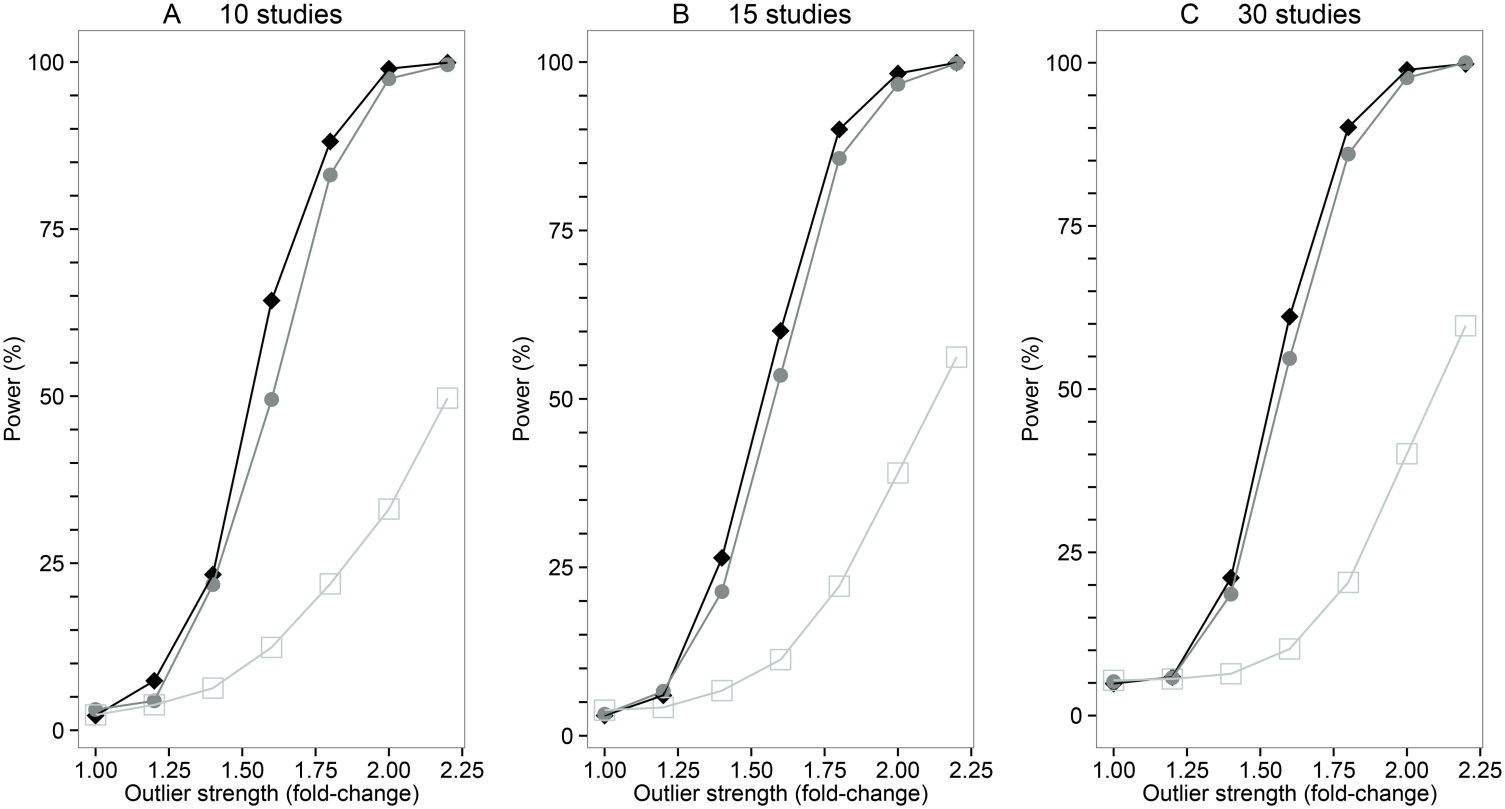
The power of the *M* statistic to detect systematic outlier studies. A power analysis of the *M* statistics for meta-analysis scenarios with varying numbers of studies and variants. The three panels show (from left to right) simulations for 10, 15 and 30 studies; 50, 25 and 10 variant simulations are shown by filled diamonds, filled circles, or open squares respectively. Each data point represents a meta-analysis simulation with 1,000 replicates, where an outlier study was assigned genetic effects that are x-fold stronger than the effects assigned to the remaining studies showing typical effects. Effect sizes for variants in the studies showing typical effects were allocated from an L—shaped distribution (S2 Table) whilst effect sizes for variants in the outlier study were calculated as a multiple of the typical effect size. For example, effect sizes for variants in an outlier study 2-fold-stronger than studies showing typical effects would be computed as (2 x ({0.04, 0.12, 0.2, 0.28, 0.4}, σ = 0.10).

Simulations were then performed to further assess the power of the *M* statistic to detect outlier studies included in a meta-analysis on a background of random heterogeneity. Table 1 shows the results from simulations where a single outlier study was included in the meta-analysis that showed a random pattern of association (i.e. the *β* coefficients for the *V* variants in the outlier study were sampled from a Φ(*β* = 0, *σ* = 0.10) distribution i.e. fold-change = zero). The power of *M* to identify the “null” outlier study increased with the number of variants but there was little impact on power varying the number of studies in the meta-analysis. We then examined scenarios where an outlier study in a meta-analysis was selected to show systematically stronger effects than the other participating studies (Fig 2). Again the power of *M* statistic to detect the outlier study increased with the number of variants included in the meta-analysis. Varying the number of studies in the meta-analysis had relatively little impact on the power to detect systematic outliers. Similarly, the power of *M* statistic to diagnose an outlier study showing systematically weaker effects than other participating studies increased with the number of variants interrogated in the meta-analysis. We also studied the impact of the background level of heterogeneity on power; this showed that it is easier to identify outlier studies with the *M* statistic if the average level of heterogeneity is low (S2 Fig).

### Detecting systematic between-study heterogeneity in a meta-analysis of coronary artery disease

The CARDIoGRAMplusC4D consortium has recently reported a GWAS meta-analysis of 60,801 CAD cases and 123,504 controls assembled from 48 studies [3]. Participants had been recruited from several ancestry groups (African American, Hispanic American, East Asian, South Asian, Middle Eastern and European). The CAD cases included patients with clinical diagnoses of myocardial infarction with or without ST-elevation, other acute coronary syndromes or chronic stable angina, as well as patients who had undergone a revascularization procedure or had angiographic evidence of stenosis (>50%) in at least 1 coronary vessel. The majority of the studies recruited CAD cases retrospectively (i.e. prevalent cases), the other prospective studies included a mixture of incident and prevalent disease. The controls included population samples who were unscreened for CAD (e.g. the UK 1958 Birth Cohort and National Blood Service controls genotyped as part of the Welcome Trust Case Control Consortium [12]) in addition to samples from volunteers with no personal history of coronary disease or individuals who had undergone coronary angiography but had no radiological evidence of vessel stenosis. Various GWAS SNP arrays had been genotyped by the studies so genotype imputation to the 1000 genomes phase 1, version 3 haplotype training set was used to facilitate the meta-analysis by maximizing the available mapping information.

In an additive-effects-only association analysis, 46 discrete CAD loci surpassed the conventional genome-wide significance threshold (i.e. *P* < 5 × 10^−8^). Variants within the 46 loci were mostly well imputed with 82% of the variants having an imputation quality score > 0.9. A lead variant (i.e. the variant with the smallest p-value) for each of these loci was selected for aggregate heterogeneity analysis, 35 of these variants showed some degree of between-study effect size heterogeneity (i.e. *I*^*2*^ > 0) (S3 Table). The 46 lead variants were in linkage equilibrium with each other.

**Fig 3:**
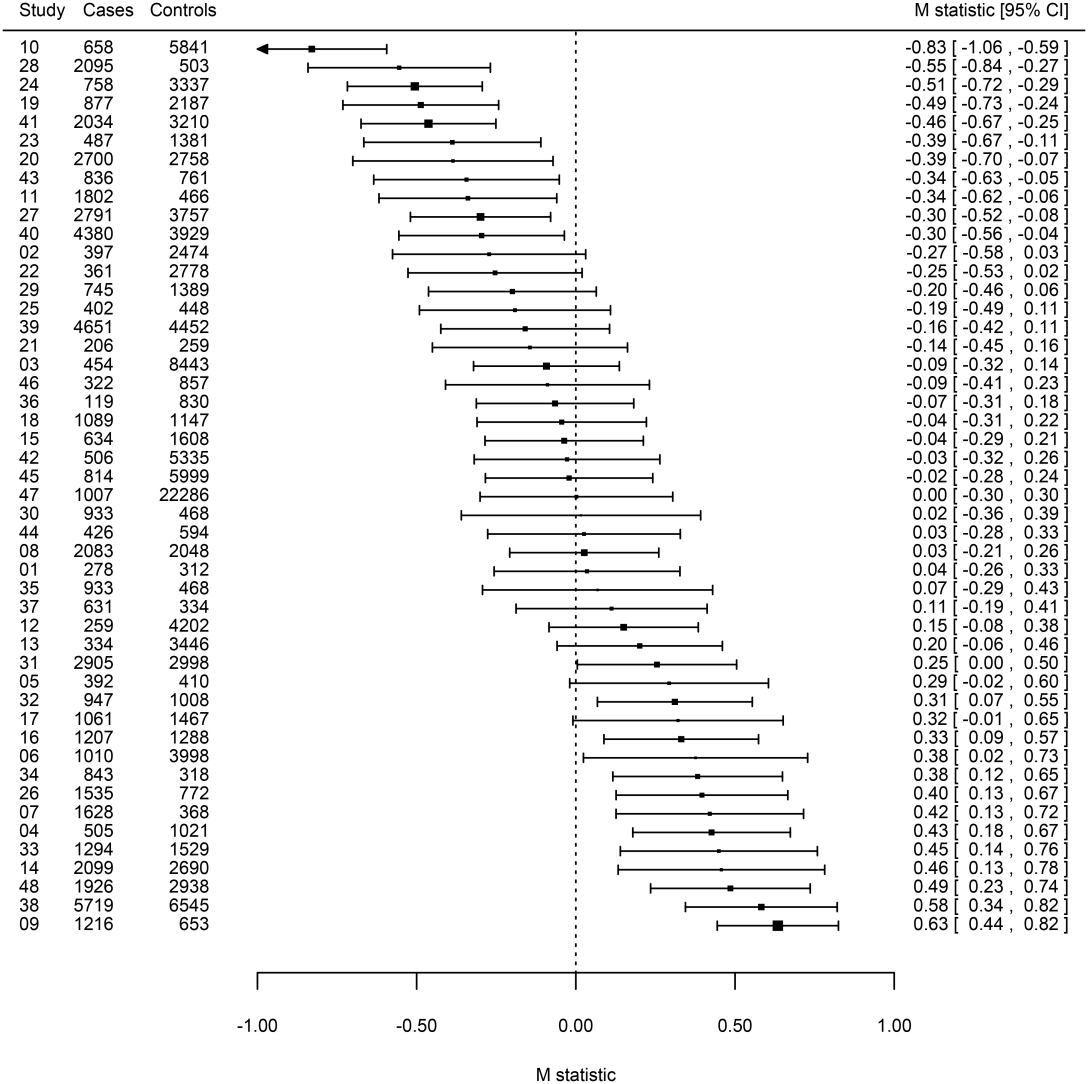
Forest plot of *M* statistics summarizing systematic patterns of heterogeneity among studies in the CARDIOGRAMplusC4D GWAS meta-analysis. Sorted *M* statistics are presented for individual studies represented by filled squares with their 95% confidence intervals shown by horizontal lines; the sizes of the squares are proportional to each studies’ inverse-variance weighting. Studies showing weaker (*M* < 0) than average genetic effects can be distinguished from those showing stronger (*M* > 0) than average effects.

Inspection of the *M* statistics for the 48 studies suggested that some studies showed systematic differences from the average genetic effect (Fig 3 and S4 Table). Notably, studies 9, 38 and 48 showed significantly stronger effects than average (Bonferroni corrected p-values < 0.05) while studies 10, 19, 24 and 28 showed significantly weaker effects (Bonferroni corrected p-values < 0.05). An inverse-variance weighted meta-analysis of the *M* statistics revealed substantial variability in the average effect across studies (*I*^*2*^ = 85.9%) (Fig 3). In an attempt to resolve underlying design factors that contributed to this systematic between-study heterogeneity pattern, we applied a random-effects meta-regression method [13] to the *M* statistics. We examined three potential sources of systematic heterogeneity that might have influenced the CARDIoGRAMplusC4D meta-analysis 1) ancestry, 2) family-history and 3) age-of-onset of disease (S5 Table). The participating studies had been independently commissioned and designed with overlapping disease case ascertainment criteria; accordingly we assigned the studies into earlier-onset (≤ 55 years) and later-onset of disease groups and flagged studies that ascertained cases with a positive family-history of disease (S5 Table).

**Fig 4:**
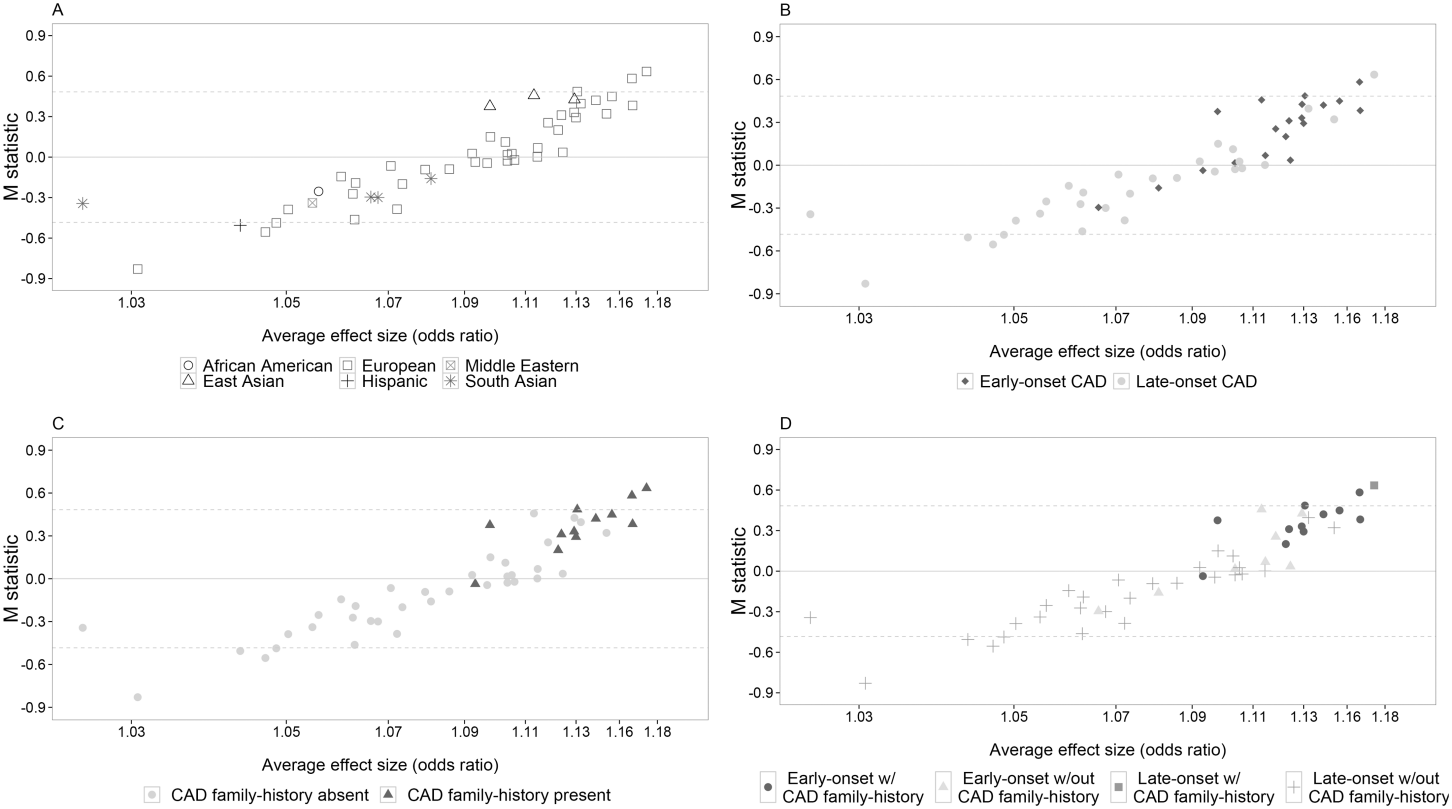
Heterogeneity in the CARDIoGRAMplusC4D meta-analysis can be explained by differences in age of CAD onset, family history and ancestry. *M* statistics for each study in the CARDIoGRAMplusC4D meta-analysis (Y- axis) are plotted against the average variant effect size (expressed as odds ratios) (X-axis) in each study. Panel A shows the ancestry of each study, panel B distinguishes early-onset from late-onset studies and panel C identifies studies ascertained with a positive family history of coronary artery disease. Panel D is a composite plot showing the degree of genetic enrichment among the studies in the meta-analysis, which ranged from non-enriched (late-onset studies without a positive family history of coronary artery disease) to doubly enriched (early-onset studies with a positive family history of coronary artery disease). The dashed lines indicate the Bonferroni corrected 5% significance threshold (*M* = ±0.483) to allow for multiple testing of 48 studies.

A meta-regression of the *M* statistics with ancestry coded into 6 groups (African and Hispanic American, South and East Asian, Middle Eastern and European) suggested that some of the variability in average effect size could be explained by ancestry (*F*_*5*, *42*_ = 2.52, *P* = 0.044) (Fig 4A). The 3 East Asian studies collectively appear to show stronger than average effects when compared with all other ancestry groups (*F*_*1*, *46*_ = 4.75, *P* = 0.034). There was no evidence that the average effects for the 38 European studies (*F*_*1*, *46*_ = 1.24, *P* = 0.271) or the 4 South Asian studies (*F*_*1*, *46*_ = 2.99, *P* = 0.090) were systematically different.

Meta-regressions of the *M* statistics suggested that studies that included early-onset cases of disease (*F*_*1*, *46*_ = 20.65, *P* = 0.00004) or included a family-history of CAD in the ascertainment scheme (*F*_*1*, *46*_ = 29.49, *P* = 2.0 × 10^−6^) showed systematically stronger than average effects (Fig 4B–4D). Finally, a multiple meta-regression analysis of East Asian ancestry, early-onset and family-history of disease showed that these factors jointly explained a significant proportion of the systematic between-study variation of average effect size (*F*_*3*, *44*_ = 13.91, *P* = 1.6 × 10^−6^; adjusted *R*^*2*^ = 53.2%) (Table 2). Additional factors examined as potential contributors to the systematic between-study differences observed included: imputation quality, genotype call rate, Hardy Weinberg equilibrium thresholds, percentage of myocardial infarction cases and case-control ratio. Their contribution to between-study variation of average effect size was negligible.

**Table 2:** Meta-regression of *M* statistics in the CARDIoGRAMplusC4D GWAS meta-analysis.

Variable	β	SE(β)	p-value
East Asian ancestry	0.348	0.172	0.049
early-onset	0.117	0.105	0.268
family history	0.409	0.111	0.001

The CARDIoGRAMplusC4D consortium studied an extended list of independently associated variants that define additional discrete loci based upon false discovery rate (FDR) criteria [3](S6 Table). These variants incremented the heritability explained over that conferred by GWAS-significant loci and might offer greater insights into heterogeneity patterns in these data. We therefore repeated the *M* statistic analysis with 214 variants (*P* < 0.00005, FDR < 5%), which confirmed the presence of systematic heterogeneity patterns in the 1000 genomes meta-analysis (S3 Fig) as well as flagging individual outlier studies (S4 Fig). Four studies, that showed insignificant outlier patterns with 46 GWAS-significant variants showed significant evidence in this analysis of FDR variants (S7 Table) and three studies that were outliers in the GWAS 46 are now insignificant. A meta-regression confirmed that East Asian ancestry, early-onset and family-history showed systematically stronger than average effects (*F*_*3*, *44*_ = 9.47, *P* = 0.0001; adjusted *R*^*2*^ = 44.8%) with family-history as the most important predictor of systematic heterogeneity in this dataset (S8 Table).

To compare our *M* analysis with a conventional single-variant strategy, we re-examined the set of GWAS-significant variants in a series of meta-regressions of three joint predictors, East Asian ancestry, early-onset and family-history. After correction for multiple testing of 46 variants, one variant (rs2891168) detected evidence of stronger associations with early-onset and family-history (*F*_*3*, *44*_ = 6.71, *P* = 0.0008; adjusted *R*^*2*^ = 44.3%) and another variant (rs6689306) showed stronger associations with East Asian ancestry (*F*_*3*, *44*_ = 7.69, *P* = 0.0003; adjusted *R*^*2*^ = 71.5%) (S5 and S6 Figs, S9 Table).

## Discussion

We present here a novel statistical approach that integrates information across multiple variants to explore background patterns of systematic between-study heterogeneity in genetic association meta-analyses. Although we have focused on examples drawn from case-control analysis where genetic association statistics have been computed by logistic regression, the method is equally applicable to other normally distributed regression statistics e.g. linear regression analysis of quantitative genetic associations. We hypothesised that design features such as ascertainment criteria for disease cases or genetic ancestry might induce genetic heterogeneity in a meta-analysis. If these design features systematically reduce the average effect size in some of the studies participating in the meta-analysis, then the overall power to detect genetic signals will be reduced. This is an important consideration, since genetic effects for CD-CV are typically small in magnitude requiring very large sample sizes for reliable detection; there is strong pressure to undertake increasingly large meta-analyses. As meta-analysis consortia expand to attain larger sample sizes, the risk that they will become increasingly diverse in terms of underlying design features must surely increase.

Analytic and Monte Carlo simulations demonstrate the potential of the proposed *M* statistic to detect systematic patterns of between-study heterogeneity. These calculations were based on a specified uniform level of heterogeneity for each variant and showed that the conventional approach to detecting heterogeneity (e.g. Cochran’s *Q* statistic) is relatively underpowered to detect systematic patterns. To maximize the power of detecting systematic heterogeneity patterns, we recommend analysing as many independently (i.e. in linkage equilibrium) and strongly associated variants as possible. In the future it would be interesting to extend the *M* approach including variants in linkage disequilibrium (LD) as this development might further enhance its power. It is anticipated that lead variants will show varying levels of heterogeneity, indeed several are likely to show little or no statistical evidence of heterogeneity (i.e. *I*^*2*^ < 25%). Such variants do though include some information relevant to detecting systematic weaker or stronger effects than average so we recommend that all firmly associated lead variants are included in the calculation of *M* statistics. Our simulations also assumed equal weightings for each contributing study, we anticipate that outlier studies with larger sample sizes (and thus weightings) will be prominent and outliers with small weightings are likely to be obscure. We also found that the background level of heterogeneity influences the power to detect outlier studies, panels of strongly associated variants that individually show moderate levels of heterogeneity (25% < *I*^*2*^ < 50%) are well suited to this approach.

We tested our new methods on data assembled for the CARDIoGRAMplusC4D GWAS meta-analysis of CAD risk [3]. Although there was marked heterogeneity of effect sizes across the participating studies (Fig 3), all studies showed positive associations with coronary disease risk (Fig 4) and thus made useful contributions to the overall discovery GWAS objective. Meta-regression of the *M* statistics revealed patterns of systematic heterogeneity that were linked to specific design features, East Asian ancestry, age-of-onset of disease and family-history. The latter two features are predicted by the multifactorial threshold model [5] to induce genetic enrichment [14]. Of note, 50 years ago the early-onset of coronary disease was recognised as a potent risk factor increasing sibling recurrence risks six-fold [15]. Although the magnitudes of the enrichment of average genetic effect size were quite modest (14% for East Asian ancestry, 15% for family-history, 11% for early-onset), we estimate that this could reduce the required sample size of cases and controls to detect an associated locus by up to 50%. Population genetic diversity, differences in the underlying rates of CAD and the relative contribution of individual risk factors, as well as lower use of preventive therapies in East Asia versus Europe (and other regions) may contribute to the enriched genetic signal observed in East Asian studies [16, 17]. A follow-up meta-regression analysis of individual variants confirmed the role of ancestry, age—of-onset and family-history as significant predictors of systematic heterogeneity. Meta-regression of multiple potential explanatory factors inevitably carries a multiple statistical testing burden, and our present results should be interpreted as an exploration of the substantial systematic heterogeneity patterning evident in Fig 4. The *M* statistic approach is advantaged over conventional single-variant methods in that information across multiple variants is aggregated reducing the dimensionality of the multiple comparison problem. Finally, we were unable to detect any systematic heterogeneity patterning attributable to the proportion of CAD cases suffering a myocardial infarction confirming the findings of the CARDIoGRAMplusC4D consortium [3].

There are several potential sources of heterogeneity that might affect genetic association meta-analysis studies. Controls for a common disease might be drawn from unscreened population samples in some studies or screened for the disease and filtered in other studies, this is predicted to dilute genetic signals in studies using population controls [18]. Environmental risk factor profiles might vary from study to study so disease cases sampled from a relatively low risk population would tend to be genetically enriched. Varying levels of LD can also induce heterogeneity [19], a situation that is particularly important for meta-analyses involving multiple ancestry groups where the lead variant is a tagging rather than the causal variant. For example, African ancestry populations typically show more haplotype diversity and lower levels of LD across the genome than European or in turn East Asian populations [20]. Thus in a multi-ethnic meta-analysis, signals detected by tagging SNPs could show systematic weaker (in low LD populations) or stronger (in high LD populations) effects that could be detected by the *M* statistic approach.

Given the momentum of the GWAS approach to identify more and more loci with improved genotype imputation training sets [21], it is inevitable that increasingly large and potentially diverse meta-analysis projects will be conceived. For diseases and traits with multiple known genetic signals, there is now an opportunity to assess the respective contributions of participating studies in newly commissioned meta-analyses. Outlier studies flagged with discrepant *M* statistics, particularly those with weaker than average effects, can be reviewed as part of the routine quality control of GWAS meta-analysis in case there are design or analytic issues that need attention to maximize power. For design issues that might be difficult to resolve, it would be useful to assess the power of performing meta-analysis in the presence and absence of the studies with discrepant *M* statistics. Following the final meta-analysis, meta-regression of *M* statistics including variants tagging previously known as well as newly discovered loci can be used to explore potential design features that might show systematic aggregate effects that are obscured in heterogeneity analyses of individual loci, and influence future study design.

## Materials and methods

### Background

Random-effects meta-analysis is a statistical procedure originally devised by epidemiologists to integrate summary information from multiple independent yet related interventional studies to estimate two parameters, Θ, the average treatment effect across the contributing studies and τ^2^, the extent of inter-study variability (or heterogeneity) in the treatment effects [22]. The effects evident in each study are assumed to be have been sampled from a normal distribution with two additive variance components, random within-study error σ^2^ and between-study variation (i.e. heterogeneity) τ^2^, so that *y*_*s*_, the measured effect in the *s*^th^ study, can be modeled by: *y*_*s*_ = Θ + ε_*s*_ + u where ε_*s*_ ~ Φ(0, σ^2^_*s*_), u ~ Φ(0, τ^2^) and Φ denotes the cumulative probability distribution function of a normal random variable.

The first step in the analysis is to estimate the magnitude of τ^2^, which can be undertaken by several algorithms [22]. This is followed by an inverse-variance weighted (i.e. $1/\sqrt{\left({\hat{\tau }}^{2}+\sigma_{s}^{2}\right)}$), least squares estimation of the average treatment effect (Θ) (which ignores the study-specific random effects) and its associated standard error (*E*, the “standard error of the prediction”).

Standardized predicted random effects (*SPRE*) can then be calculated for each of the studies as $SPRE=\left(y_{s}-\theta \right)/\sqrt{{\hat{\tau }}^{2}+\sigma_{s}^{2}-E^{2}}$; these are precision-weighted, standard normally distributed statistics (i.e. *SPRE* ~ *N*(0, 1)) that summarize the extent and the direction that individual studies differ from the average treatment effect. If there is no evidence of heterogeneity of effects (i.e. *τ*^2^ = 0), then the *SPREs* are identical to standardized predicted fixed effects derived from a fixed-effects meta-analysis. A normal probability plot of the *SPRE* statistics provides a convenient visual way to detect outlier studies that might be unduly influencing the estimate of the average treatment effect that complements inspection of a Forest plot.

### A novel multi-variant heterogeneity statistic

Consider now a genetic association meta-analysis project comprising *S* studies with summary-level results for *V* genetic variants. Genetic effect-sizes (and their standard errors) have been estimated in each study by regression modelling to substitute for the treatment effects described above. Assume that the variants selected for heterogeneity analysis are truly associated with the disease or quantitative trait and are in linkage equilibrium (i.e. uncorrelated) with each other. Informative variants could include 1) published variants that have previously shown strong evidence of association or 2) the lead variants at GWAS-significant loci in a post-hoc heterogeneity analysis. The genetic effects need to be synchronized so that the average Θ estimates for each variant are positive (i.e. all average effects are “in the same direction” consistent with higher disease risks or levels of a quantitative trait); this can be achieved by judicious “flipping” of the regression coefficients submitted by participating studies.

For each of *V* variants, estimate τ^2^, Θ and E using the random-effects procedure detailed above and calculate and store *SPRE* statistics for each of *S* studies in a regular array *SPRE*_*sv*_ (S1 Methods).

Subsequently, a “mean” aggregate statistic can be calculated that summarizes between-study heterogeneity across multiple genetic variants:

$$M_{s}=\;\frac{1}{V}\sum_{v=1}^{V}SPRE_{sv}.$$

Under the assumption that *M*_*s*_ is a linear combination of *V* mutually independent, standard normal random variables, then *M*_*s*_ will be normally distributed with expectation (i.e. mean) 0 and variance 1/*V* (S2 Methods). Positive or negative values of *M*_*s*_ indicate that the study shows systematically larger or smaller genetic effects than the average effect, statistically significant deviations are found where |*M*_*s*_| exceeds an appropriate threshold; we corrected for multiple testing of *S* studies by applying the Bonferroni procedure to control the family-wise error rate (FWER) < 0.05. We developed a Stata script (*getmstatistic)* based on the *metareg* programme [23] to calculate *M*_*s*_ statistics using τ^2^ estimates derived from the restricted maximum log-likelihood (REML) method. Additionally, an R package (*Rgetmstatistic)* for *getmstatistic* has been developed.

### Power calculations

To support the use of this newly proposed statistic, we examined the impact of several systematic heterogeneity scenarios by means of analytic and Monte-Carlo simulation-based power analyses. We first compared our new method with Cochran’s *Q* statistic, a widely used and computationally simple method to screen for between-study heterogeneity [7, 8]. *Q* statistics approximate a chi-squared distribution in large samples [24], for each scenario non-centrality parameters were equated with calculated *Q* statistics (i.e. treating *Q* as a log likelihood ratio statistic [25]; [26]). The non-centrality parameter was then used in standard chi-squared power calculations ([26]), with an allowance for multiple testing of *V* variants by applying Bonferroni’s correction to control the family-wide error rate (FWER) to 5%. Denote the power to detect heterogeneity in a meta-analysis of the *v*^*th*^ variant by **ω**_***v***_, then the overall power to detect at least one heterogeneous variant is

$$\omega =1-\prod_{1}^{V}1-\omega_{v}$$

To calculate the analytic power of *M*, it is convenient to introduce a Wald statistic (*M*^*2*^), the squared-standardized *M* statistic i.e. $M^{2}={\left(\frac{M}{SE_{M}}\right)}^{2}$ where SEM=(1V)12, which is approximately chi-squared distributed on 1 degree of freedom. *M*^*2*^ can then be substitute for the non-centrality parameter in standard chi-squared power calculations [26] allowing for multiple testing of *S* studies by applying Bonferroni’s correction to control the family-wide error rate (FWER) to 5%. Denote the power (**ω**) to detect heterogeneity in a meta-analysis for the *s*^*th*^ study by **ω**_***s***_, then the overall power to detect at least one heterogeneous variant is

$$\omega =1-\prod_{1}^{S}1-\omega_{s}$$

The above analytic power calculations were performed using scripts and in-built procedures in Stata 10.1.

We also carried out Monte-Carlo simulations for scenarios where a systematic heterogeneity pattern is superimposed on a background random heterogeneity pattern, this allows for the possibility that real world heterogeneity patterns have multiple sources and complexity. These simulations allowed the comparison of the distributions of empirical with asymptotic p-values, with empirical p-values calculated using the (*r*+1)/(*n*+1) estimator [27] where *r* represents the rank of the simulated statistic and *n* the total number of replicates in the simulation exercise.

### Meta-regression of M statistics

To explore the impact of design features on the magnitude of *M* that vary between individual studies participating in a meta-analysis, we apply a random-effects meta-regression procedure (*metareg*) in Stata 10.1 to regress towards the average deviation of the observed effects of studies. This analysis is based upon study-specific *M* statistics to summarize the studies’ overall deviation from the average effect with precision weighting (i.e. $1/SE_{M_{s}}$ for the *s*^*th*^ study—see S2 Methods) to allow for differing sample sizes in different studies.

### Ethics statement

The studies contributing to the CARDIoGRAMplusC4D study were approved by the ethics committees of the respective medical faculties, and informed consent was obtained from all participants. Summary genetic association data were anonymously meta-analysed and reported here.

### Web resources

Software to calculate *M* statistics is available at the following url: *getmstatistic*, https://magosil86.github.io/getmstatistic

### Supplemental data

Supplemental data includes the membership of the CARDIoGRAMplusC4D Consortium, six figures and nine tables.

## Supporting information

S1 Fig Empirical and theoretical distributions of the *M* statistic. Monte—Carlo simulations comparing the empirical (histograms) and theoretical frequency distributions (density plots) of *M* statistics. The four panels show (from A to D) simulations for 2, 10, 25 and 50 variants over 10,000 replicates.
(TIF)

S2 Fig Power to detect systematic outlier studies in the presence of variable background heterogeneity. A power analysis of *M* involving Monte-Carlo GWAS meta-analysis scenarios varying the level of background heterogeneity (I^2^ from 8.89% to 86.2%). Each data point represents a simulation based on 15 studies and 50 variants. All studies were equally weighted (i.e. SE = 0.08). Effect sizes for variants in the studies showing typical effects were allocated from an L—shaped distribution (S2 Table) whilst effect sizes for variants in the outlier study were calculated as a multiple of the typical effect size (i.e. 1.80 x ({0.04, 0.12, 0.2, 0.28, 0.4}, σ = 0.10) to model a 1.8-fold stronger-than-typical outlier study.
(TIF)

S3 Fig Forest plot of *M* statistics computed across 214 loci (FDR < 5%) summarizing systematic patterns of heterogeneity among studies in the CARDIOGRAMplusC4D GWAS meta-analysis. Sorted *M* statistics are presented for individual studies represented by filled squares with their 95% confidence intervals shown by horizontal lines; the sizes of the squares are proportional to each studies’ inverse-variance weighting. Studies showing weaker (*M* < 0) than average genetic effects can be distinguished from those showing stronger (*M* > 0) than average effects.
(TIF)

S4 Fig A scatterplot of *M* statistics computed across 214 loci (FDR < 5%). *M* statistics for each study in the CARDIoGRAMplusC4D meta-analysis (Y- axis) are plotted against the average variant effect size (expressed as odds ratios) (X-axis) in each study. A colour gradient was employed to highlight the distribution of *M* statistics. The dashed lines indicate the Bonferroni corrected 5% significance threshold (*M* = ±0.224) to allow for multiple testing of 48 studies.
(TIF)

S5 Fig Forest plot of effect-sizes (log odds ratios) at rs2891168 (chromosome 9) highlighting locus specific heterogeneity among studies in the CARDIOGRAMplusC4D GWAS meta-analysis (I^2^ = 57.69%). Sorted odds ratios are presented for individual studies represented by filled squares with their 95% confidence intervals shown by horizontal lines; the sizes of the squares are proportional to each studies’ inverse-variance weighting. A filled diamond represents the summary effect-size.
(TIF)

S6 Fig Forest plot of effect-sizes (log odds ratios) at rs6689306 (chromosome 1) highlighting locus specific heterogeneity among studies in the CARDIOGRAMplusC4D GWAS meta-analysis (I^2^ = 40.57%). Sorted odds ratios are presented for individual studies represented by filled squares with their 95% confidence intervals shown by horizontal lines; the sizes of the squares are proportional to each studies’ inverse-variance weighting. A filled diamond represents the summary effect-size.
(TIF)

S1 Text Membership, affiliation and supporting references for the CARDIoGRAMplusC4D Consortium. (DOCX)

S1 Methods Computing *SPREs*. (DOCX)

S2 Methods Computing *M* statistics. (DOCX)

S1 Table A comparison of the theoretical and empirical null distributions for *M*. (XLSX)

S2 Table Number of variants included in each effect size bin for 3 simulation scenarios. (XLSX)

S3 Table Lead variants at 46 loci selected for computation of *M* in the CARDIoGRAMplusC4D GWAS meta-analysis. (XLSX)

S4 Table Studies showing substantial systematic patterns of heterogeneity in the CARDIoGRAMplusC4D GWAS meta-analysis at threshold, alpha = 0.05. (XLSX)

S5 Table Ancestry, age-of-disease onset and family-history of disease grouping for CARDIoGRAMplusC4D studies. (XLSX)

S6 Table Dataset employed in computing *M* statistics for 214 FDR ≤ 5% lead variants in the CARDIoGRAMplusC4D GWAS meta-analysis. (XLSX)

S7 Table Studies showing systematic heterogeneity patterns across 214 FDR ≤ 5% lead variants in the CARDIoGRAMplusC4D GWAS meta-analysis at threshold alpha = 0.05. (XLSX)

S8 Table Meta-regression of *M* statistics computed across 214 FDR ≤ 5% lead variants in the CARDIoGRAMplusC4D GWAS meta-analysis. (XLSX)

S9 Table Meta-regression of effect-sizes in rs2891168 and rs6689306 employing East Asian ancestry, early-age-of-disease onset and family history of CAD as covariates. (XLSX)
