# Supplementary figures and images for: Identifying systematic heterogeneity patterns in genetic association meta-analysis studies
Source: PLoS Genet. 2017 May 1;13(5):e1006755. doi: 10.1371/journal.pgen.1006755 (PMC5432194; doi:10.1371/journal.pgen.1006755)

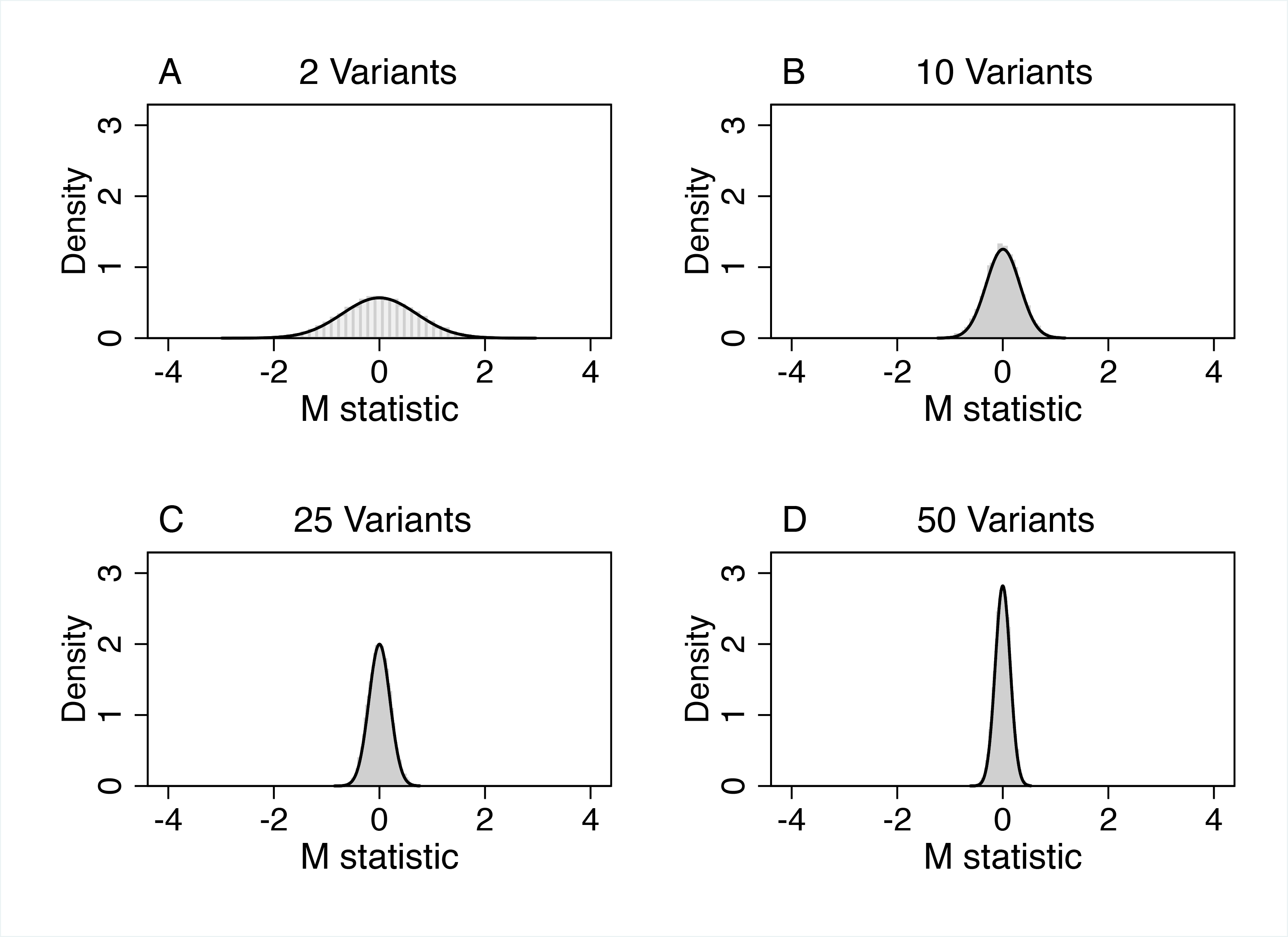

Supplement: S1 Fig — Monte—Carlo simulations comparing the empirical (histograms) and theoretical frequency distributions (density plots) of M statistics. The four panels show (from A to D) simulations for 2, 10, 25 and 50 variants over 10,000 replicates. (TIF) [file pgen.1006755.s001.tif]

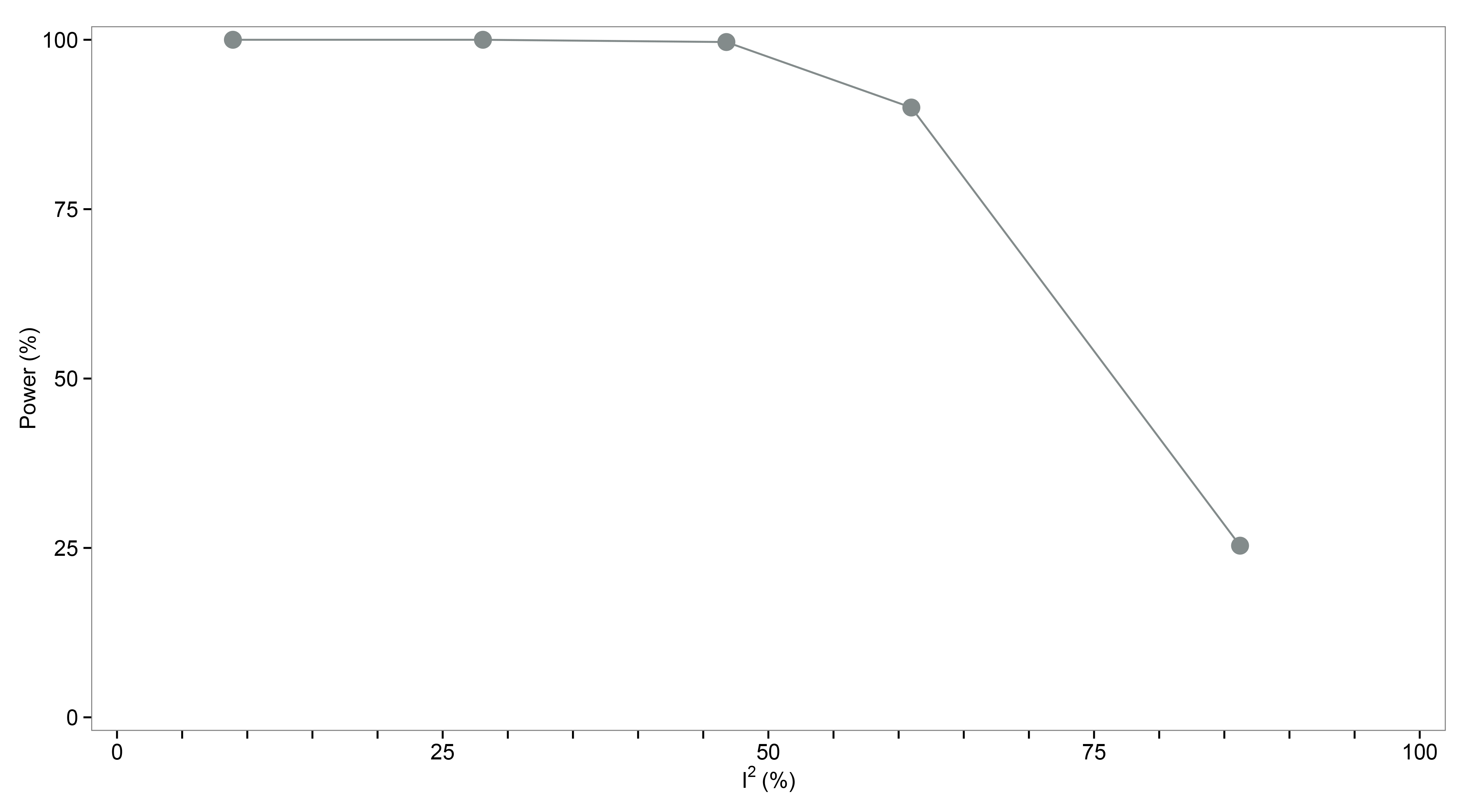

Supplement: S2 Fig — A power analysis of M involving Monte-Carlo GWAS meta-analysis scenarios varying the level of background heterogeneity (I2 from 8.89% to 86.2%). Each data point represents a simulation based on 15 studies and 50 variants. All studies were equally weighted (i.e. SE = 0.08). Effect sizes for variants in the studies showing typical effects were allocated from an L—shaped distribution (S2 Table) whilst effect sizes for variants in the outlier study were calculated as a multiple of the typical effect size (i.e. 1.80 x ({0.04, 0.12, 0.2, 0.28, 0.4}, σ = 0.10) to model a 1.8-fold stronger-than-typical outlier study. (TIF) [file pgen.1006755.s002.tif]

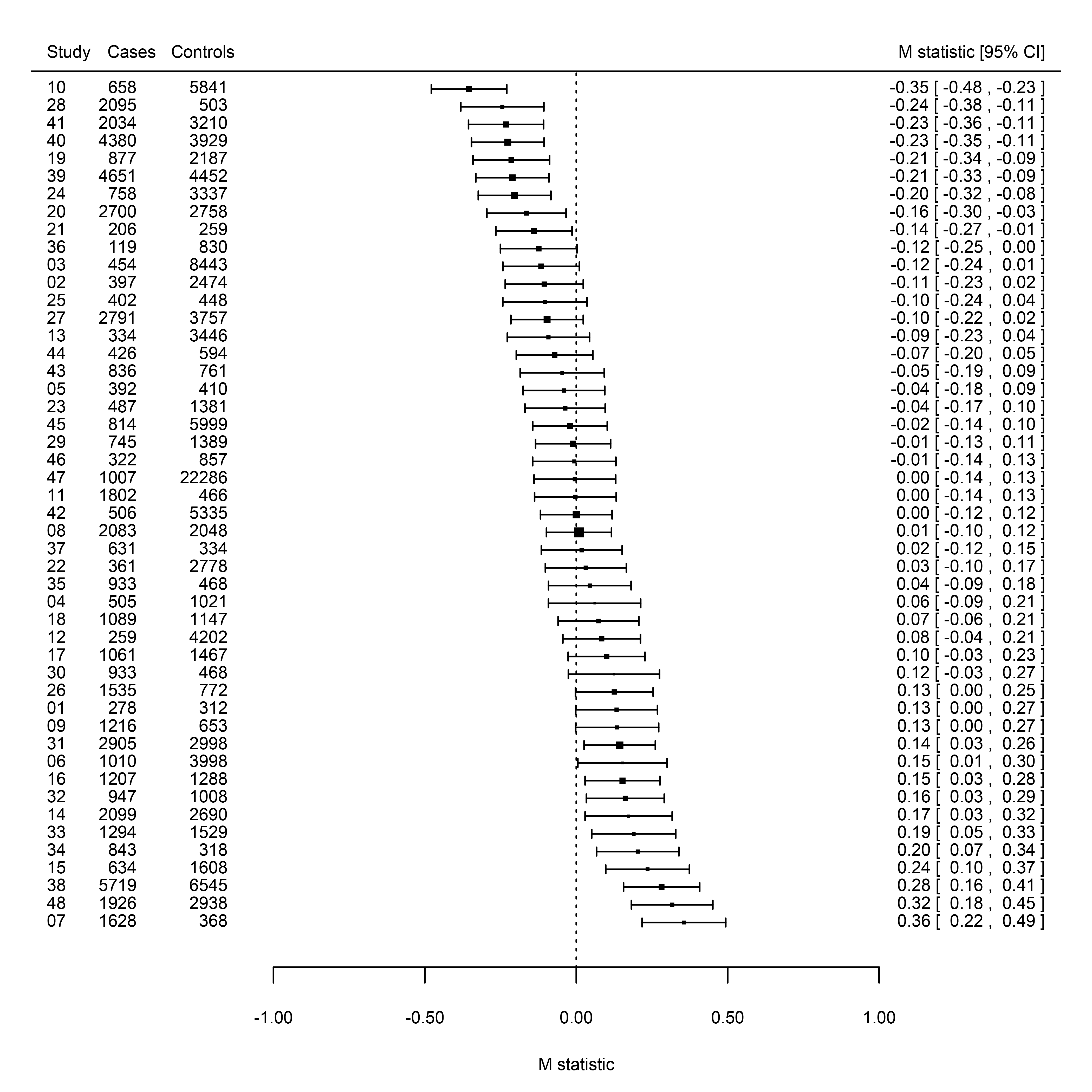

Supplement: S3 Fig — Sorted M statistics are presented for individual studies represented by filled squares with their 95% confidence intervals shown by horizontal lines; the sizes of the squares are proportional to each studies’ inverse-variance weighting. Studies showing weaker (M < 0) than average genetic effects can be distinguished from those showing stronger (M > 0) than average effects. (TIF) [file pgen.1006755.s003.tif]

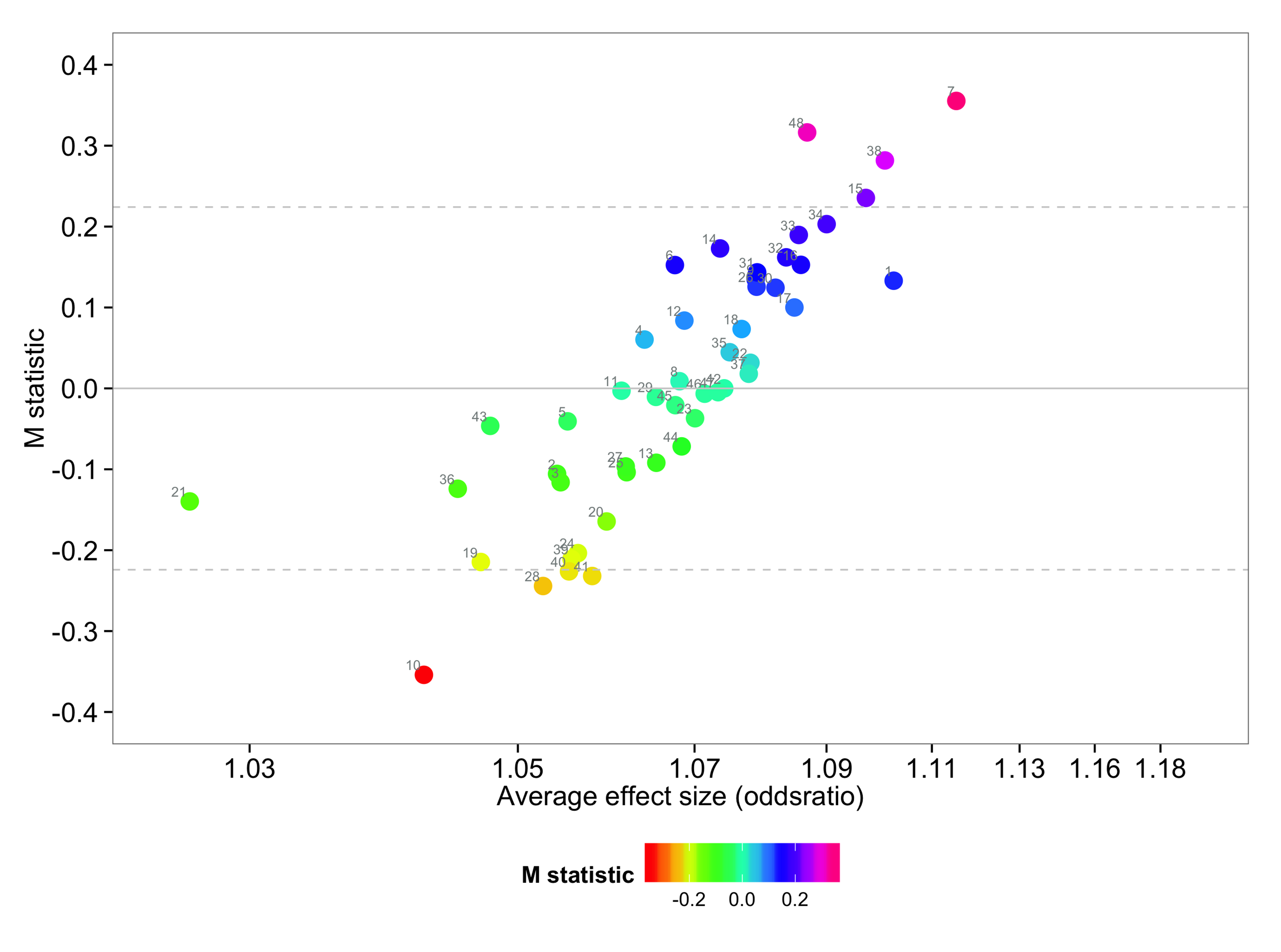

Supplement: S4 Fig — M statistics for each study in the CARDIoGRAMplusC4D meta-analysis (Y- axis) are plotted against the average variant effect size (expressed as odds ratios) (X-axis) in each study. A colour gradient was employed to highlight the distribution of M statistics. The dashed lines indicate the Bonferroni corrected 5% significance threshold (M = ±0.224) to allow for multiple testing of 48 studies. (TIF) [file pgen.1006755.s004.tif]

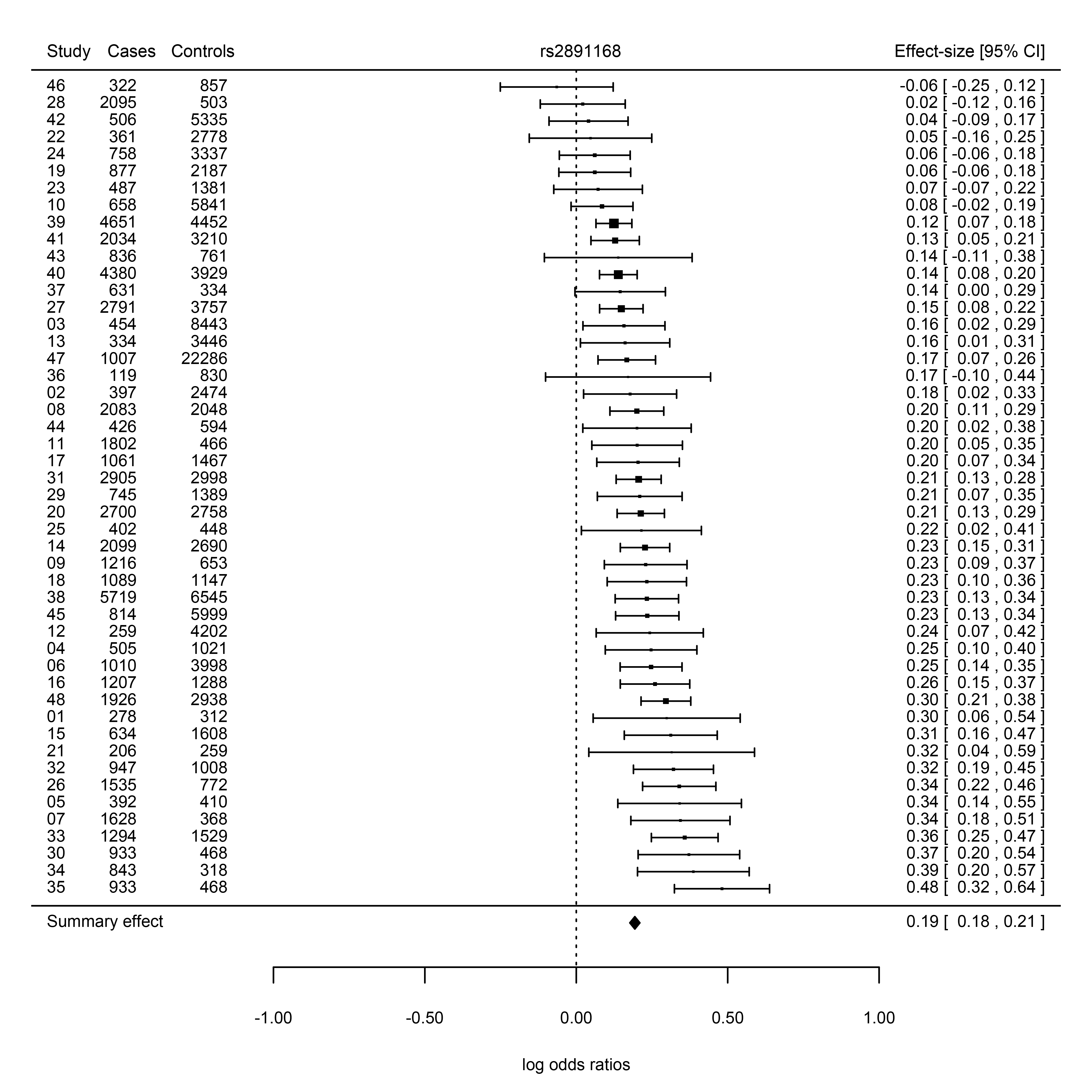

Supplement: S5 Fig — Sorted odds ratios are presented for individual studies represented by filled squares with their 95% confidence intervals shown by horizontal lines; the sizes of the squares are proportional to each studies’ inverse-variance weighting. A filled diamond represents the summary effect-size. (TIF) [file pgen.1006755.s005.tif]

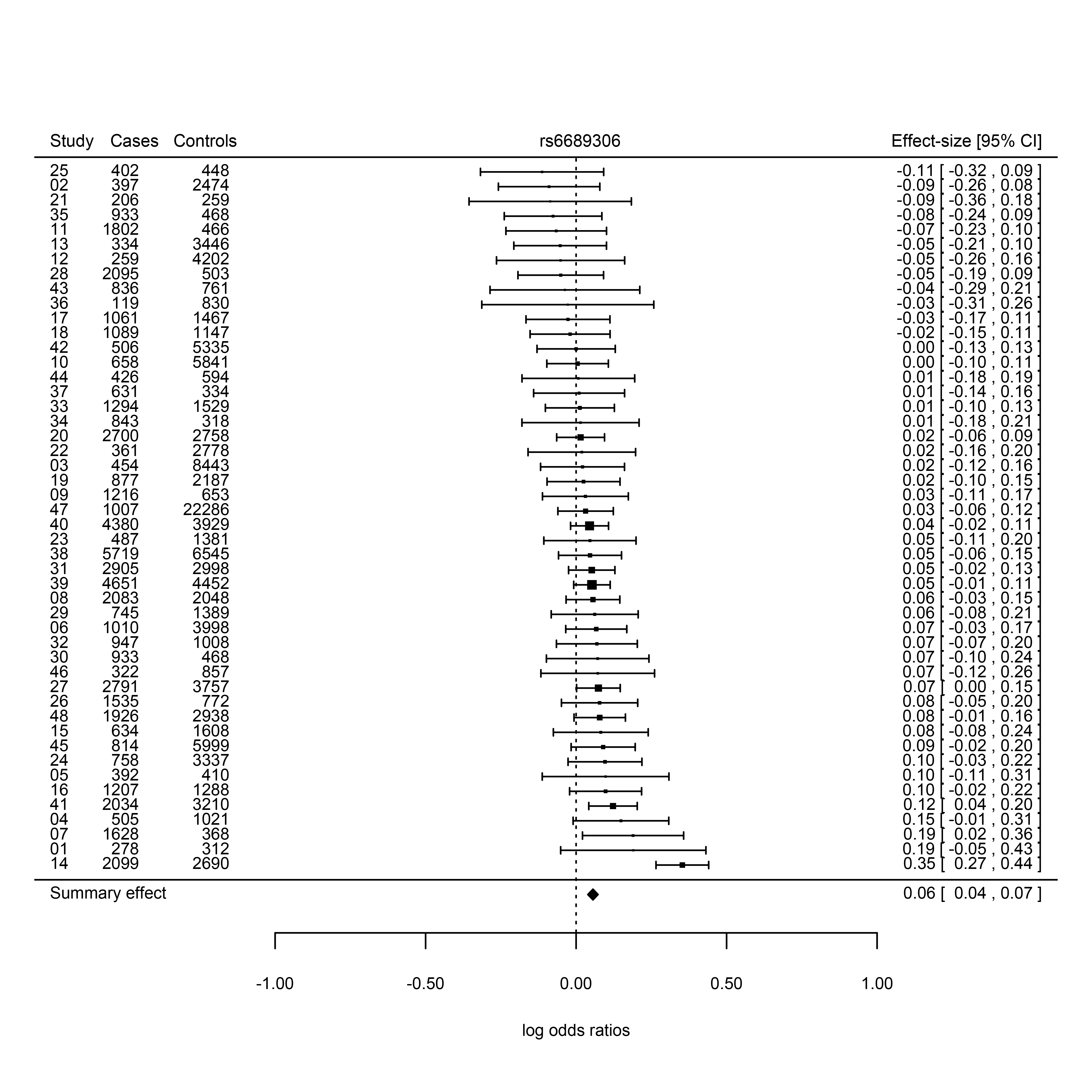

Supplement: S6 Fig — Sorted odds ratios are presented for individual studies represented by filled squares with their 95% confidence intervals shown by horizontal lines; the sizes of the squares are proportional to each studies’ inverse-variance weighting. A filled diamond represents the summary effect-size. (TIF) [file pgen.1006755.s006.tif]
