# Supplementary material for: Identifying systematic heterogeneity patterns in genetic association meta-analysis studies
Source: PLoS Genet. 2017 May 1;13(5):e1006755. doi: 10.1371/journal.pgen.1006755 (PMC5432194; doi:10.1371/journal.pgen.1006755)
Supplement: S1 Text — (DOCX) [file pgen.1006755.s007.docx]

**S1 Text**

**Membership, affiliation and supporting references for the CARDIoGRAMplusC4D Consortium**

Majid Nikpay[1], Anuj Goel[2,3], Hong-Hee Won[4,5,6], Leanne M Hall[7], Christina Willenborg[8,9], Stavroula Kanoni[10], Danish Saleheen[11,12], Theodosios Kyriakou[2,3], Christopher P Nelson[7,13], Jemma C Hopewell[14], Thomas R Webb[7], Lingyao Zeng[15,16], Abbas Dehghan[17], Maris Alver[18,19], Sebastian M Armasu[20], Kirsi Auro[21,22], Andrew Bjonnes[23,24], Daniel I Chasman[25,26], Shufeng Chen[27], Ian Ford[28], Nora Franceschini[29], Christian Gieger[16,30], Stefan Gustafsson[31], Jie Huang[32], Shih-Jen Hwang[33,34], Yun Kyong Kim[35], Marcus E Kleber[36], King Wai Lau[14], Yingchang Lu[37,38], Xiangfeng Lu[27], Leo P Lyytikäinen[39,40], Evelin Mihailov[18], Alanna Morrison[41], Natalia Pervjakova[18,21,22], Liming Qu[42], Lynda M Rose[25], Elias Salfati[43], Richa Saxena[23,24], Markus Scholz[44,45], Albert V Smith[46,47], Emmi Tikkanen[48,49], Andre Uitterlinden[17], Xueli Yang[27], Weihua Zhang[50,51], Wei Zhao[11], Mariza de Andrade[20], Paul S de Vries[17], Natalie R van Zuydam[3,52], Sonia S Anand[53], Lars Bertram[54], Frank Beutner[45,55], George Dedoussis[56], Dominique Gauguier[57], Alison H Goodall[13,58], Omri Gottesman[37], Marc Haber[59], Bok-Ghee Han[35], Jianfeng Huang[60], Thorsten Kessler[61], Inke R König[9,62], Lars Lannfelt[63], Wolfgang Lieb[64], Lars Lind[65], Cecilia M Lindgren[3,4], Maisa Lokki[66], Patrik K Magnusson[67], Narinder Mehra[68], Thomas Meitinger[16,69], Andrew P Morris[3,70], Markku S Nieminen[71], Nancy L Pedersen[67], Annette Peters[16,30], Loukianos S Rallidis[72], Svati H Shah[73], Juha Sinisalo[71], Kathleen E Stirrups[10,74], Stella Trompet[75,76], Laiyuan Wang[27,77], Diego Ardissino[78], Eric Boerwinkle[41,79], Ingrid B Borecki[80], Erwin P Bottinger[37], Julie E Buring[25,26], John C Chambers[50,51,81], Rory Collins[14], L Adrienne Cupples[33,82], John Danesh[32,83], Roberto Elosua[84], Stephen E Epstein[85], Tõnu Esko[18,86,87], Mary F Feitosa[80], Oscar H Franco[17], Maria G Franzosi[88], Christopher B Granger[73], Dongfeng Gu[27], Vilmundur Gudnason[46,47], Alistair S Hall[89], Anders Hamsten[90], Tamara B Harris[91], Stanley L Hazen[92], Christian Hengstenberg[15,16], Albert Hofman[17], Erik Ingelsson[3,31], Carlos Iribarren[93], J Wouter Jukema[75,94,95], Pekka J Karhunen[39,96], Bong-Jo Kim[35], Jaspal S Kooner[51,81,97], Iftikhar J Kullo[98], Terho Lehtimäki[39,40], Ruth J Loos[37,38,99], Olle Melander[100], Andres Metspalu[18,19], Winfried März[36,101,102], Colin N Palmer[52], Markus Perola[18,21,22], Thomas Quertermous[43], Daniel J Rader[103,104], Paul M Ridker[25,26], Samuli Ripatti[32,48,49], Robert Roberts[105], Veikko Salomaa[106], Dharambir K Sanghera[107,108,109], Stephen M Schwartz[110,111], Udo Seedorf[112], Alexandre F Stewart[1], David J Stott[113], Joachim Thiery[45,114], Pierre A Zalloua[59,115], Christopher J O'Donnell[33,82,116], Muredach P Reilly[104], Themistocles L Assimes[43], John R Thompson[117], Jeanette Erdmann[8,9], Robert Clarke[14], Hugh Watkins[2,3], Sekar Kathiresan[4,5,6], Ruth McPherson[1], Panos Deloukas[10,118], Heribert Schunkert[15,16], Nilesh J Samani[7,13], Martin Farrall[2,3]

Affiliations

1. Ruddy Canadian Cardiovascular Genetics Centre University of Ottawa Heart Institute, Ottawa, Canada

2. Division of Cardiovascular Medicine, Radcliffe Department of Medicine, University of Oxford, Oxford, UK

3. Wellcome Trust Centre for Human Genetics, University of Oxford, Oxford, UK

4. Broad Institute of the Massachusetts Institute of Technology and Harvard University, Cambridge, Massachusetts, USA

5. Cardiovascular Research Center and Center for Human Genetic Research, Massachusetts General Hospital, Boston, Massachusetts, USA

6. Department of Medicine, Harvard Medical School, Boston, Massachusetts, USA

7. Department of Cardiovascular Sciences, University of Leicester, Leicester, UK

8. Institut für Integrative und Experimentelle Genomik, Universität zu Lübeck, Lübeck, Germany

9. DZHK (German Research Center for Cardiovascular Research) partner site Hamburg–Lübeck–Kiel, Lübeck, Germany

10. William Harvey Research Institute, Barts and the London School of Medicine and Dentistry, Queen Mary University of London, London, UK

11. Perelman School of Medicine, University of Pennsylvania, Philadelphia, Pennsylvania, USA

12. Center for Noncommunicable Diseases, Karachi, Pakistan

13. NIHR Leicester Cardiovascular Biomedical Research Unit, Glenfield Hospital, Leicester, UK

14. CTSU, Nuffield Department of Population Health, University of Oxford, Oxford, UK

15. Deutsches Herzzentrum München, Technische Universität München, München, Germany

16. DZHK (German Centre for Cardiovascular Research), partner site Munich Heart Alliance, Munich, Germany

17. Department Of Epidemiology, Erasmus University Medical center, Rotterdam, The Netherlands

18. Estonian Genome Center, University of Tartu, Tartu, Estonia

19. Institute of Molecular and Cell Biology, Tartu, Estonia

20. Division of Biomedical Statistics and Informatics, Department of Health Sciences Research, Mayo Clinic, Rochester, Minnesota, USA

21. Department of Health, National Institute for Health and Welfare, Helsinki, Finland

22. University of Helsinki, Institute for Molecular Medicine, Finland (FIMM) and Diabetes & Obesity Research Program, Helsinki, Finland

23. Center for Human Genetic Research, Boston, Massachusetts, USA

24. Massachusetts General Hospital, Boston, Massachusetts, USA

25. Division of Preventive Medicine, Brigham and Women's Hospital, Boston, Massachusetts, USA

26. Harvard Medical School, Boston, Massachusetts, USA

27. State Key Laboratory of Cardiovascular Disease, Fuwai Hospital, National Center of Cardiovascular Diseases, Chinese Academy of Medical Sciences and Peking Union Medical College, Beijing, China

28. Robertson Center for Biostatistics, University of Glasgow, Glasgow, UK

29. Department of Epidemiology, Gillings School of Global Public Health, University of North Carolina, Chapel Hill, North Carolina, USA

30. Institute of Epidemiology II, Helmholtz Zentrum München, German Research Center for Environmental Health, Neuherberg, Germany

31. Department of Medical Sciences, Molecular Epidemiology and Science for Life Laboratory, Uppsala University, Uppsala, Sweden

32. Wellcome Trust Sanger Institute, Hinxton, Cambridge, UK

33. National Heart, Lung, and Blood Institute's Framingham Heart Study, Framingham, Massachusetts, USA

34. Department of Biostatistics, Boston University School of Public Health, Boston, Massachusetts, USA

35. Center for Genome Science, Korea National Institute of Health, Chungcheongbuk-do, Korea

36. Vth Department of Medicine (Nephrology, Hypertensiology, Endocrinology, Diabetology, Rheumatology), Medical Faculty of Mannheim, University of Heidelberg, Mannheim, Germany

37. The Charles Bronfman Institute for Personalized Medicine, The Icahn School of Medicine at Mount Sinai, New York, New York, USA

38. The Genetics of Obesity and Related Metabolic Traits Program, The Icahn School of Medicine at Mount Sinai, New York, New York, USA

39. Department of Clinical Chemistry, Fimlab Laboratories, Tampere, Finland

40. Department of Clinical Chemistry, University of Tampere School of Medicine, Tampere, Finland

41. Human Genetics Center, School of Public Health, The University of Texas Health Science Center at Houston, Houston, Texas, USA

42. Department of Biostatistics and Epidemiology, University of Pennsylvania, Philadelphia, Pennsylvania, USA

43. Stanford Cardiovascular Institute and the Division of Cardiovascular Medicine, Stanford University, Stanford, California, USA

44. Institute for Medical Informatics, Statistics and Epidemiology, Medical Faculty, University of Leipzig, Leipzig, Germany

45. LIFE Research Center of Civilization Diseases, Leipzig, Germany

46. Icelandic Heart Association, Kopavogur, Iceland

47. Faculty of Medicine, University of Iceland, Reykjavik, Iceland

48. Hjelt Institute, University of Helsinki, Helsinki, Finland

49. Institute for Molecular Medicine Finland FIMM, University of Helsinki, Helsinki, Finland

50. Department of Epidemiology and Biostatistics, Imperial College London, London, UK

51. Department of Cardiology, Ealing Hospital NHS Trust, Middlesex, UK

52. Medical Research Institute, University of Dundee, Dundee, UK

53. Department of Medicine, McMaster University, Hamilton, Ontario, Canada

54. Platform for Genome Analytics, Institutes of Neurogenetics & Integrative and Experimental Genomics, University of Lübeck, Lübeck, Germany

55. Heart Center Leipzig, Cardiology, University of Leipzig, Leipzig, Germany

56. Department of Dietetics-Nutrition, Harokopio University, Athens, Greece

57. INSERM, UMRS1138, Centre de Recherche des Cordeliers, Paris, France

58. Department of Cardiovascular Sciences, University of Leicester, Glenfield Hospital, Leicester, UK

59. Lebanese American University, School of Medicine, Beirut, Lebanon

60. Hypertension Division, Fuwai Hospital, National Center For Cardiovascular Diseases, Chinese Academy of Medical Sciences and Peking Union Medical College, Beijing, China

61. Klinikum rechts der Isar, and Deutsches Herzzentrum München, München, Germany

62. Institut für Medizinische Biometrie und Statistik, Universität zu Lübeck, Lübeck, Germany

63. Department of Public Health and Caring Sciences, Geriatrics, Uppsala University, Uppsala, Sweden

64. Institut für Epidemiologie, Christian-Albrechts Universität zu Kiel, Kiel, Germany

65. Department of Medical Sciences, Cardiovascular Epidemiology, Uppsala University, Uppsala, Sweden

66. Transplantation Laboratory, Haartman Institute, University of Helsinki, Helsinki, Finland

67. Department of Medical Epidemiology and Biostatistics, Karolinska Institutet, Stockholm, Sweden

68. All India Institute of Medical Sciences, New Delhi, India

69. Institut für Humangenetik, Helmholtz Zentrum München, German Research Center for Environmental Health, Neuherberg, Germany

70. Department of Biostatistics, University of Liverpool, Liverpool, UK

71. Department of Medicine, Department of Cardiology, Helsinki University Central Hospital, Helsinki, Finland

72. Second Department of Cardiology, Attikon Hospital, School of Medicine, University of Athens, Athens, Greece

73. Department of Medicine, Duke University Medical Center, Durham, North Carolina, USA

74. Department of Haematology, University of Cambridge, Cambridge, UK

75. Department of Cardiology, Leiden University Medical Center, Leiden, The Netherlands

76. Department of Gerontology and Geriatrics, Leiden University Medical Center, Leiden, The Netherlands

77. National Human Genome Center at Beijing, Beijing, China

78. Division of Cardiology, Azienda Ospedaliero-Universitaria di Parma, Parma, Italy

79. Human Genome Sequencing Center, Baylor College of Medicine, Houston, Texas, USA

80. Department of Genetics, Washington University School of Medicine, St. Louis, Missouri, USA

81. Imperial College Healthcare NHS Trust, London, UK

82. National Heart, Lung and Blood Institute Division of Intramural Research, Bethesda, Maryland, USA

83. University of Cambridge, Cambridge, UK

84. Grupo de Epidemiología y Genética Cardiovascular, Institut Hospital del Mar d’Investigacions Mèdiques (IMIM), Barcelona, Spain

85. MedStar Heart and Vascular Institute, MedStar Washington Hospital Center, Washington, DC, USA

86. Division of Endocrinology and Basic and Translational Obesity Research, Boston Children's Hospital, Boston, Massachusetts, USA

87. Department of Genetics, Harvard Medical School, Boston, Massachusetts, USA

88. Department of Cardiovascular Research, IRCCS Istituto di Ricerche Farmacologiche Mario Negri, Milano, Italy

89. Leeds Institute of Genetics, Health and Therapeutics, University of Leeds, Leeds, UK

90. Cardiovascular Genetics and Genomics Group, Atherosclerosis Research Unit, Department of Medicine Solna, Karolinska Institutet, Stockholm, Sweden

91. Laboratory of Epidemiology, Demography, and Biometry, National Institute on Aging, National Institutes of Health, Bethesda, Maryland, USA

92. Cleveland Clinic, Cleveland, Ohio, USA

93. Kaiser Permanente Division of Research, Oakland, California, USA

94. Durrer Center for Cardiogenetic Research, Amsterdam, The Netherlands

95. Interuniversity Cardiology Institute of the Netherlands, Utrecht, The Netherlands

96. Department of Forensic Medicine, University of Tampere School of Medicine, Tampere, Finland

97. Cardiovascular Science, National Heart and Lung Institute, Imperial College London, London, UK

98. Division of Cardiovascular Diseases, Department of Medicine, Mayo Clinic, Rochester, Minnesota, USA

99. The Mindich Child Health and Development Institute, The Icahn School of Medicine at Mount Sinai, New York, New York, USA

100. Department of Clinical Sciences, Diabetes and Endocrinology, Lund University, University Hospital Malmö, Malmö, Sweden

101. Synlab Academy, Synlab Services GmbH, Mannheim, Germany

102. Clinical Institute of Medical and Chemical Laboratory Diagnostics, Medical University of Graz, Graz, Austria

103. Department of Genetics, Perelman School of Medicine at the University of Pennsylvania, Philadelphia, Pennsylvania, USA

104. Cardiovascular Institute, Perelman School of Medicine at the University of Pennsylvania, Philadelphia, Pennsylvania, USA

105. University of Ottawa Heart Institute, Ottawa, Canada

106. Department of Chronic Disease Prevention, National Institute for Health and Welfare, Helsinki, Finland

107. Department of Pediatrics, College of Medicine, University of Oklahoma Health Sciences Center, Oklahoma City, Oklahoma, USA

108. Department of Pharmaceutical Sciences, College of Pharmacy, University of Oklahoma Health Sciences Center, Oklahoma City, Oklahoma, USA

109. Oklahoma Center for Neuroscience, Oklahoma City, Oklahoma, USA

110. Public Health Sciences Division, Fred Hutchinson Cancer Research Center, Seattle, Washington, USA

111. Department of Epidemiology, University of Washington, Seattle, Washington, USA

112. Department of Prosthetic Dentistry, Center for Dental and Oral Medicine, University Medical Center Hamburg-Eppendorf, Hamburg, Germany

113. Institute of Cardiovascular and Medical Sciences, Faculty of Medicine, University of Glasgow, Glasgow, UK

114. Institute for Laboratory Medicine, Clinical Chemistry and Molecular Diagnostics, University Hospital Leipzig, Medical Faculty, Leipzig, Germany

115. Harvard School of Public Health, Boston, Massachusetts, USA

116. Cardiology Division, Massachusetts General Hospital, Boston, Massachusetts, USA

117. Department of Health Sciences, University of Leicester, Leicester, UK

118. Princess Al-Jawhara Al-Brahim Centre of Excellence in Research of Hereditary Disorders (PACER-HD), King Abdulaziz University, Jeddah, Saudi Arabia

**Supporting references**

1. Schunkert, H., Konig, I.R., Kathiresan, S., Reilly, M.P., Assimes, T.L., Holm, H., Preuss, M., Stewart, A.F., Barbalic, M., Gieger, C., et al. (2011). Large-scale association analysis identifies 13 new susceptibility loci for coronary artery disease. Nat. Genet. *43*, 333-338.

2. Harris, T.B., Launer, L.J., Eiriksdottir, G., Kjartansson, O., Jonsson, P.V., Sigurdsson, G., Thorgeirsson, G., Aspelund, T., Garcia, M.E., Cotch, M.F., et al. (2007). Age, Gene/Environment Susceptibility-Reykjavik Study: multidisciplinary applied phenomics. Am. J. Epidemiol. *165*, 1076-1087.

3. Dehghan, A., Bis, J.C., White, C.C., Smith, A.V., Morrison, A.C., Cupples, L.A., Trompet, S., Chasman, D.I., Lumley, T., Volker, U., et al. (2016). Genome-Wide Association Study for Incident Myocardial Infarction and Coronary Heart Disease in Prospective Cohort Studies: The CHARGE Consortium. PLoS One *11*, e0144997.

4. (1989). The Atherosclerosis Risk in Communities (ARIC) Study: design and objectives. The ARIC investigators. Am. J. Epidemiol. *129*, 687-702.

5. Lu, X., Wang, L., Chen, S., He, L., Yang, X., Shi, Y., Cheng, J., Zhang, L., Gu, C.C., Huang, J., et al. (2012). Genome-wide association study in Han Chinese identifies four new susceptibility loci for coronary artery disease. Nat. Genet. *44*, 890-894.

6. Samani, N.J., Erdmann, J., Hall, A.S., Hengstenberg, C., Mangino, M., Mayer, B., Dixon, R.J., Meitinger, T., Braund, P., Wichmann, H.E., et al. (2007). Genomewide association analysis of coronary artery disease. N. Engl. J. Med. *357*, 443-453.

7. Davies, R.W., Wells, G.A., Stewart, A.F., Erdmann, J., Shah, S.H., Ferguson, J.F., Hall, A.S., Anand, S.S., Burnett, M.S., Epstein, S.E., et al. (2012). A genome-wide association study for coronary artery disease identifies a novel susceptibility locus in the major histocompatibility complex. Circ. Cardiovasc. Genet. *5*, 217-225.

8. Vaara, S., Nieminen, M.S., Lokki, M.L., Perola, M., Pussinen, P.J., Allonen, J., Parkkonen, O., and Sinisalo, J. (2012). Cohort Profile: the Corogene study. Int. J. Epidemiol. *41*, 1265-1271.

9. Consortium, C.A.D., Deloukas, P., Kanoni, S., Willenborg, C., Farrall, M., Assimes, T.L., Thompson, J.R., Ingelsson, E., Saleheen, D., Erdmann, J., et al. (2013). Large-scale association analysis identifies new risk loci for coronary artery disease. Nat. Genet. *45*, 25-33.

10. Leitsalu, L., Haller, T., Esko, T., Tammesoo, M.L., Alavere, H., Snieder, H., Perola, M., Ng, P.C., Magi, R., Milani, L., et al. (2015). Cohort Profile: Estonian Biobank of the Estonian Genome Center, University of Tartu. Int. J. Epidemiol. *44*, 1137-1147.

11. Hager, J., Kamatani, Y., Cazier, J.B., Youhanna, S., Ghassibe-Sabbagh, M., Platt, D.E., Abchee, A.B., Romanos, J., Khazen, G., Othman, R., et al. (2012). Genome-wide association study in a Lebanese cohort confirms PHACTR1 as a major determinant of coronary artery stenosis. PLoS One *7*, e38663.

12. Higgins, M., Province, M., Heiss, G., Eckfeldt, J., Ellison, R.C., Folsom, A.R., Rao, D.C., Sprafka, J.M., and Williams, R. (1996). NHLBI Family Heart Study: objectives and design. Am. J. Epidemiol. *143*, 1219-1228.

13. Lee, J.Y., Lee, B.S., Shin, D.J., Woo Park, K., Shin, Y.A., Joong Kim, K., Heo, L., Young Lee, J., Kyoung Kim, Y., Jin Kim, Y., et al. (2013). A genome-wide association study of a coronary artery disease risk variant. J. Hum. Genet. *58*, 120-126.

14. Erdmann, J., Willenborg, C., Nahrstaedt, J., Preuss, M., Konig, I.R., Baumert, J., Linsel-Nitschke, P., Gieger, C., Tennstedt, S., Belcredi, P., et al. (2011). Genome-wide association study identifies a new locus for coronary artery disease on chromosome 10p11.23. Eur. Heart J. *32*, 158-168.

15. Nikpay, M., Goel, A., Won, H.H., Hall, L.M., Willenborg, C., Kanoni, S., Saleheen, D., Kyriakou, T., Nelson, C.P., Hopewell, J.C., et al. (2015). A comprehensive 1,000 Genomes-based genome-wide association meta-analysis of coronary artery disease. Nat Genet 47, 1121-1130.

16. Morris, A.D., Boyle, D.I., MacAlpine, R., Emslie-Smith, A., Jung, R.T., Newton, R.W., and MacDonald, T.M. (1997). The diabetes audit and research in Tayside Scotland (DARTS) study: electronic record linkage to create a diabetes register. DARTS/MEMO Collaboration. BMJ *315*, 524-528.

17. Tyynela, P., Goebeler, S., Ilveskoski, E., Mikkelsson, J., Perola, M., Loytonen, M., and Karhunen, P.J. (2009). Birthplace predicts risk for prehospital sudden cardiac death in middle-aged men who migrated to metropolitan area: The Helsinki Sudden Death Study. Ann. Med. *41*, 57-65.

18. Gottesman, O., Kuivaniemi, H., Tromp, G., Faucett, W.A., Li, R., Manolio, T.A., Sanderson, S.C., Kannry, J., Zinberg, R., Basford, M.A., et al. (2013). The Electronic Medical Records and Genomics (eMERGE) Network: past, present, and future. Genet. Med. *15*, 761-771.

19. Coronary Artery Disease Genetics, C. (2011). A genome-wide association study in Europeans and South Asians identifies five new loci for coronary artery disease. Nat. Genet. *43*, 339-344.

20. Winkelmann, B.R., Marz, W., Boehm, B.O., Zotz, R., Hager, J., Hellstern, P., Senges, J., and Group, L.S. (2001). Rationale and design of the LURIC study--a resource for functional genomics, pharmacogenomics and long-term prognosis of cardiovascular disease. Pharmacogenomics *2*, S1-73.

21. Reilly, M.P., Li, M., He, J., Ferguson, J.F., Stylianou, I.M., Mehta, N.N., Burnett, M.S., Devaney, J.M., Knouff, C.W., Thompson, J.R., et al. (2011). Identification of ADAMTS7 as a novel locus for coronary atherosclerosis and association of ABO with myocardial infarction in the presence of coronary atherosclerosis: two genome-wide association studies. Lancet *377*, 383-392.

22. Myocardial Infarction Genetics, C., Kathiresan, S., Voight, B.F., Purcell, S., Musunuru, K., Ardissino, D., Mannucci, P.M., Anand, S., Engert, J.C., Samani, N.J., et al. (2009). Genome-wide association of early-onset myocardial infarction with single nucleotide polymorphisms and copy number variants. Nat. Genet. *41*, 334-341.

23. Lind, L., Fors, N., Hall, J., Marttala, K., and Stenborg, A. (2005). A comparison of three different methods to evaluate endothelium-dependent vasodilation in the elderly: the Prospective Investigation of the Vasculature in Uppsala Seniors (PIVUS) study. Arterioscler. Thromb. Vasc. Biol. *25*, 2368-2375.

24. Borodulin, K., Vartiainen, E., Peltonen, M., Jousilahti, P., Juolevi, A., Laatikainen, T., Mannisto, S., Salomaa, V., Sundvall, J., and Puska, P. (2015). Forty-year trends in cardiovascular risk factors in Finland. Eur. J. Public Health *25*, 539-546.

25. Saleheen, D., Zaidi, M., Rasheed, A., Ahmad, U., Hakeem, A., Murtaza, M., Kayani, W., Faruqui, A., Kundi, A., Zaman, K.S., et al. (2009). The Pakistan Risk of Myocardial Infarction Study: a resource for the study of genetic, lifestyle and other determinants of myocardial infarction in South Asia. Eur. J. Epidemiol. *24*, 329-338.

26. Trompet, S., de Craen, A.J., Postmus, I., Ford, I., Sattar, N., Caslake, M., Stott, D.J., Buckley, B.M., Sacks, F., Devlin, J.J., et al. (2011). Replication of LDL GWAs hits in PROSPER/PHASE as validation for future (pharmaco)genetic analyses. BMC Med. Genet. *12*, 131.

27. Hofman, A., Darwish Murad, S., van Duijn, C.M., Franco, O.H., Goedegebure, A., Ikram, M.A., Klaver, C.C., Nijsten, T.E., Peeters, R.P., Stricker, B.H., et al. (2013). The Rotterdam Study: 2014 objectives and design update. Eur. J. Epidemiol. *28*, 889-926.

28. Tabassum, R., Chauhan, G., Dwivedi, O.P., Mahajan, A., Jaiswal, A., Kaur, I., Bandesh, K., Singh, T., Mathai, B.J., Pandey, Y., et al. (2013). Genome-wide association study for type 2 diabetes in Indians identifies a new susceptibility locus at 2q21. Diabetes *62*, 977-986.

29. Saxena, R., Bjonnes, A., Prescott, J., Dib, P., Natt, P., Lane, J., Lerner, M., Cooper, J.A., Ye, Y., Li, K.W., et al. (2014). Genome-wide association study identifies variants in casein kinase II (CSNK2A2) to be associated with leukocyte telomere length in a Punjabi Sikh diabetic cohort. Circ. Cardiovasc. Genet. *7*, 287-295.

30. Theodoraki, E.V., Nikopensius, T., Suhorutsenko, J., Peppes, V., Fili, P., Kolovou, G., Papamikos, V., Richter, D., Zakopoulos, N., Krjutskov, K., et al. (2010). Fibrinogen beta variants confer protection against coronary artery disease in a Greek case-control study. BMC Med. Genet. *11*, 28.

31. Hong, Y., Pedersen, N.L., Brismar, K., and de Faire, U. (1997). Genetic and environmental architecture of the features of the insulin-resistance syndrome. Am. J. Hum. Genet. *60*, 143-152.

32. Ingelsson, E., Sundstrom, J., Arnlov, J., Zethelius, B., and Lind, L. (2005). Insulin resistance and risk of congestive heart failure. JAMA *294*, 334-341.

33. Ridker, P.M., Chasman, D.I., Zee, R.Y., Parker, A., Rose, L., Cook, N.R., Buring, J.E., and Women's Genome Health Study Working, G. (2008). Rationale, design, and methodology of the Women's Genome Health Study: a genome-wide association study of more than 25,000 initially healthy american women. Clin. Chem. *54*, 249-255.
