# Supplementary material for: Identifying systematic heterogeneity patterns in genetic association meta-analysis studies
Source: PLoS Genet. 2017 May 1;13(5):e1006755. doi: 10.1371/journal.pgen.1006755 (PMC5432194; doi:10.1371/journal.pgen.1006755)
Supplement: S1 Methods — (DOCX) [file pgen.1006755.s008.docx]

**S1 Methods**

Computing *SPREs*

Consider a GWAS meta-analysis ($P),$ that includes $S$ GWAS studies $(s = 1, 2, 3, \cdots, S)$ and $V$ associated variants $(v = 1, 2, 3, \cdots, V).$ Data for each variant are analyzed using a random-effects model to estimate the average genetic effect and partition the variability in effect sizes across the studies into random sampling and heterogeneity components. Then, the standardized predicted random effect (SPRE) for the *v^th^* variant in the *s^th^* study can be calculated as:

$$SPRE_{sv}=\frac{y_{sv}- \theta_{v}}{\sqrt{\sigma_{sv}^{2}+ \tau_{v}^{2}- s_{p_{sv}}^{2}}} ,$$

where

$y_{sv}$ : genetic effect of the *v^th^* variant in the *s^th^* study

$\theta_{v}$ : average effect of the *v^th^* variant across all studies

$\sigma_{sv}^{2}$ : sampling variance for the *v^th^* variant in the *s^th^* study

$\tau_{v}^{2}$ : estimate of between-study (heterogeneity) variance for the *v^th^* variant

$s_{p_{sv}}$: standard error of prediction (fitted values) for the *v^th^* variant in the *s^th^* study excluding random effects

such that $\left( y_{sv}- \theta_{v} \right)$represents the raw residual and $\sqrt{\left( \sigma_{sv}^{2}+ \tau_{v}^{2}- s_{p_{sv}}^{2} \right)}$ the unconditional standard error of the predicted random effect[Harbord & Higgins 2008].

Ultimately, computing *SPREs* for each variant in each study will yield an array,

$$P_{S,V}= \left[ \begin{matrix} SPRE_{1,1} & SPRE_{1,2} & \cdots& SPRE_{1,v} \\ SPRE_{2,1} & SPRE_{2,2} & \cdots& SPRE_{2,v} \\ \vdots& \vdots& \ddots& \vdots\\ SPRE_{s,1} & SPRE_{s,2} & \cdots& SPRE_{s,v} \end{matrix} \right] .$$

Next, the heterogeneity information (i.e. *SPREs*) from multiple variants is aggregated by study, to diagnose outlier studies and their direction of effect (i.e. whether they consistently show stronger or weaker effects than average).

Harbord RM, & Higgins, J. P. T. Meta-regression in Stata. Stata Journal. 2008;*8*:493–519.
