# Supplementary material for: Identifying systematic heterogeneity patterns in genetic association meta-analysis studies
Source: PLoS Genet. 2017 May 1;13(5):e1006755. doi: 10.1371/journal.pgen.1006755 (PMC5432194; doi:10.1371/journal.pgen.1006755)
Supplement: S2 Methods — (DOCX) [file pgen.1006755.s009.docx]

S2 Methods

Computing *M* statistics

To compute the "mean" aggregate heterogeneity statistic, *M*, the arithmetic mean of *SPREs* within each of the $S$ studies is calculated, yielding a single *M* statistic value for each study. For the *s^th^* study, the *M* statistic value is defined as,

$$M_{s}= \frac{1}{V}\sum_{v=1}^{V} {SPRE}_{sv} .$$

Assuming that the *SPREs* of variants within each study are mutually independent standard normal random variables i.e.

$$SPRE \sim\Phi\left( 0,1 \right) ,$$

with expectation: $E\left( SPRE \right)= \mu= 0$ and variance: $Var\left( SPRE \right)= \sigma^{2} = 1^{2} = 1$ .

Then, as a sum of independent normal random variables, the *M* statistic for the *s^th^* study, with $V$ variants is also normally distributed

$$M_{s} \sim\Phi\left( 0, \frac{1}{V} \right) ,$$

with expectation: $E\left( M_{s} \right)= V \times\left( \frac{1}{V} \right)\times\mu= \mu= 0$

and variance: $Var\left( M_{s} \right)= V \times( {\frac{1}{V})}^{2} \times\sigma^{2} = (\frac{1}{V})1^{2} = \frac{1}{V}$ .

Assuming that the *V* variants in the meta-analysis represent a sample of the total population of associated variants, then $\bar{M}_{s}$ provides an estimate of the population mean with standard error[Feller 2008]

$${SE}_{\bar{M}_{s}}=\left( \frac{Var\left( {SPRE}_{s} \right)}{V} \right)^{\frac{1}{2}} .$$

In the event that a study has incomplete genotype data, SPREs can be substituted for the missing variants, such that, each missing variant is allocated the expected mean, $(\frac{1}{V}) \times0$ of the theoretical distribution of the *M* statistic, for *V* variants under the null hypothesis ($Ho$).

Feller W. An introduction to probability theory and its applications: John Wiley & Sons; 2008.
